# *Crenothrix* are major methane consumers in stratified lakes

**DOI:** 10.1038/ismej.2017.77

**Published:** 2017-06-06

**Authors:** Kirsten Oswald, Jon S Graf, Sten Littmann, Daniela Tienken, Andreas Brand, Bernhard Wehrli, Mads Albertsen, Holger Daims, Michael Wagner, Marcel MM Kuypers, Carsten J Schubert, Jana Milucka

**Affiliations:** 1 Department of Surface Waters—Research and Management, Eawag, Swiss Federal Institute of Aquatic Science and Technology, Kastanienbaum, Switzerland; 2 Institute of Biogeochemistry and Pollutant Dynamics, ETH Zurich, Department of Environmental Systems Science, Swiss Federal Institute of Technology, Zurich, Switzerland; 3 Department of Biogeochemistry, Max Planck Institute for Marine Microbiology, Bremen, Germany; 4 Department of Chemistry and Bioscience, Center for Microbial Communities, Aalborg University, Aalborg, Denmark; 5 Division of Microbial Ecology, Department of Microbiology and Ecosystem Science, Research Network Chemistry meets Microbiology, University of Vienna, Vienna, Austria

## Abstract

Methane-oxidizing bacteria represent a major biological sink for methane and are thus Earth’s natural protection against this potent greenhouse gas. Here we show that in two stratified freshwater lakes a substantial part of upward-diffusing methane was oxidized by filamentous gamma-proteobacteria related to *Crenothrix polyspora*. These filamentous bacteria have been known as contaminants of drinking water supplies since 1870, but their role in the environmental methane removal has remained unclear. While oxidizing methane, these organisms were assigned an ‘unusual’ methane monooxygenase (MMO), which was only distantly related to ‘classical’ MMO of gamma-proteobacterial methanotrophs. We now correct this assignment and show that *Crenothrix* encode a typical gamma-proteobacterial PmoA. Stable isotope labeling in combination swith single-cell imaging mass spectrometry revealed methane-dependent growth of the lacustrine *Crenothrix* with oxygen as well as under oxygen-deficient conditions. *Crenothrix* genomes encoded pathways for the respiration of oxygen as well as for the reduction of nitrate to N_2_O. The observed abundance and planktonic growth of *Crenothrix* suggest that these methanotrophs can act as a relevant biological sink for methane in stratified lakes and should be considered in the context of environmental removal of methane.

## Introduction

Freshwater lakes represent large natural sources of methane and contribute more to methane emissions than the oceans despite their comparably smaller area (Bastviken *et al.*, 2004). Highest rates of methane removal are usually measured at the oxyclines, either in the water column or in the sediment. Lake Rotsee and Lake Zug in Central Switzerland are typical examples of temperate lake systems with methane fluxes across the oxycline of 13±3 mmol and 10±3 mmol m^−2^ d^−1^, respectively (Oswald *et al.*, 2015, 2016). Both lakes are stratified, with methane-rich hypolimnia, but whereas the shallow Lake Rotsee overturns annually, the deep Lake Zug remains stratified throughout the year. In both lakes, the vast majority of the upward-diffusing methane is removed at the base of the oxycline at *in situ* oxygen concentrations in the low micromolar range (Oswald *et al.*, 2015, 2016). Methane oxidation at the oxycline was shown to be coupled to the reduction of residual or *in situ*-produced oxygen, but there were also indications for methane-oxidizing activity under oxygen-deficient conditions (Oswald *et al.*, 2015, 2016).

Abundant gamma-proteobacterial methane-oxidizing bacteria (gamma-MOB) were shown to be involved in methane removal in both lakes (Oswald *et al.*, 2015, 2016). Gamma-MOB are considered aerobes requiring oxygen for methane activation, even though some cultured representatives can perform methane oxidation under denitrifying conditions (Kits *et al.*, 2015a, 2015b). Environmentally relevant representatives of gamma-MOB in lakes and other freshwater habitats belong to the ‘classical’ genera of *Methylobacter*, *Methylomonas*, *Methylosarcina* and *Methylomicrobium* (Boschker *et al.*, 1998; Bodelier *et al.*, 2013; Oshkin *et al.*, 2015), and all possess particulate methane monooxygenase (pMMO) as the key methane-oxidizing enzyme (Bowman, 2005). In Lake Rotsee and Lake Zug, unicellular gamma-MOB represented a stable community at the oxycline. The bacteria showed rapid growth on methane as evidenced by the increase in cell abundances and the uptake of ^13^C-methane into their biomass (Oswald *et al.*, 2015, 2016).

In these studies, gamma-MOB were identified by fluorescence *in situ* hybridization using the 16S rRNA-targeted oligonucleotide probes Mgamma84+705. Interestingly however, these probes do not bind to members of a potentially important subgroup of gamma-proteobacterial MOB, the putative family *Crenothrichaceae*. Contrary to ‘classical’ MOB, these gamma-MOB are multicellular and filamentous. So far, only two of these bacteria have been documented in literature, *Crenothrix polyspora* and *Clonothrix fusca*, and both were retrieved from groundwater (Stoecker *et al.*, 2006; Vigliotta *et al.*, 2007). Sporadically, environmental occurrence of *Crenothrix* is reported in literature based on retrieved 16S rRNA or *pmoA* sequences (Dörr *et al.*, 2010; Drewniak *et al.*, 2012), but its role in methane cycling has remained unclear.

The metabolism of *Crenothrix* has been a matter of debate since its first description as ‘Brunnenfaden’ (‘a well thread’ Cohn, 1870). Initially, *Crenothrix/Clonothrix* filaments were considered to belong to the ‘iron bacteria’ due to the presence of metal particles in their sheaths (Roze, 1896; Jackson, 1902; Molisch, 1910). This belief was challenged by studies that failed to observe iron encrustation in *Crenothrix/Clonothrix* filaments (Kolk, 1938; Wolfe, 1960), and the later discovery of membrane invaginations has prompted suggestions for a methanotrophic lifestyle (Völker *et al.*, 1977). Eventually, the capacity to oxidize methane was experimentally confirmed on filaments retrieved from man-made habitats (Stoecker *et al.*, 2006; Vigliotta *et al.*, 2007). Interestingly, *C. polyspora* was reported to possess an ‘unusual’ pMMO, which was only distantly related to ‘classical’ MMO of gamma-proteobacterial methanotrophs (Stoecker *et al.*, 2006), and has now been recognized to cluster together with the ammonium monooxygenases of completely nitrifying ‘comammox’ bacteria (Daims *et al.*, 2015; van Kessel *et al.,* 2015).

Here we investigated the occurrence and involvement of these filamentous bacteria in methane oxidation at and below the oxyclines of Lake Rotsee and Lake Zug. We performed stable isotope labeling experiments followed by single-cell imaging to explore the role of these microorganisms in environmental methane cycling, and metagenomic analyses to investigate their metabolic potential with respect to aerobic and anaerobic respiration. For comparison, we also performed metagenomic analysis of a sample from Wolfenbüttel waterworks sand filter reportedly containing high proportions of *C. polyspora*.

## Materials and methods

### Geochemical profiling in Lake Rotsee

Profiling was done in October 2014 at the deepest point (16 m depth, 47°04.259‘N, 8°18.989‘E). A multi-parameter probe was used to measure photosynthetically active radiation (PAR; LI-193 Spherical Underwater Quantum Sensor, LI-COR, Lincoln, NE, USA) along with conductivity, turbidity, depth (pressure), temperature and pH (XRX 620, RBR, Ottawa, ON, Canada). Dissolved oxygen was simultaneously monitored online with normal and trace micro-optodes (types PSt1 and TOS7, Presens, Regensburg, Germany) with detection limits of 125 and 20 nm, respectively, and a response time of 7 s (Kirf *et al.*, 2014).

Water samples for dissolved methane analysis were retrieved from distinct depths with a Niskin bottle. Serum bottles (120 ml) were filled completely without bubbles or headspace through a gas-tight outlet tubing allowing water to overflow. Solid copper chloride [Cu(I)Cl] was immediately added in excess to the water samples and the bottles were crimped. Before analysis, a 30 ml headspace was set with N_2_ and after overnight equilibration methane concentrations were measured in the headspace with a gas chromatograph (GC; Agilent 6890 N, Agilent Technologies, Santa Clara, CA, USA) equipped with a Carboxen 1010 column (30 m × 0.53 mm, Supelco, Bellefonte, PA, USA) and a flame ionization detector. Methane concentrations in the water phase were back-calculated according to (Wiesenburg and Guinasso, 1979). Stable carbon isotopes of methane were determined in the same headspace by isotope ratio mass spectrometry with a trace gas instrument (T/GAS PRE CON, Micromass UK Ltd., Wilmslow, UK) coupled to a mass spectrometer (GV Instruments, Manchester, UK; Isoprime, Stockport, UK). Isotopic ratios are given in δ-notation relative to the Vienna Pee Dee Belemnite reference standard.

Oxygen, PAR, methane concentration and methane isotope profiles for the sampling campaign in October 2014 are shown in Supplementary Figure 1. Geochemical profiles from other Lake Rotsee campaigns are reported in Oswald *et al.* (2015).

### Lake Rotsee methane oxidation rates

**Figure 1:**
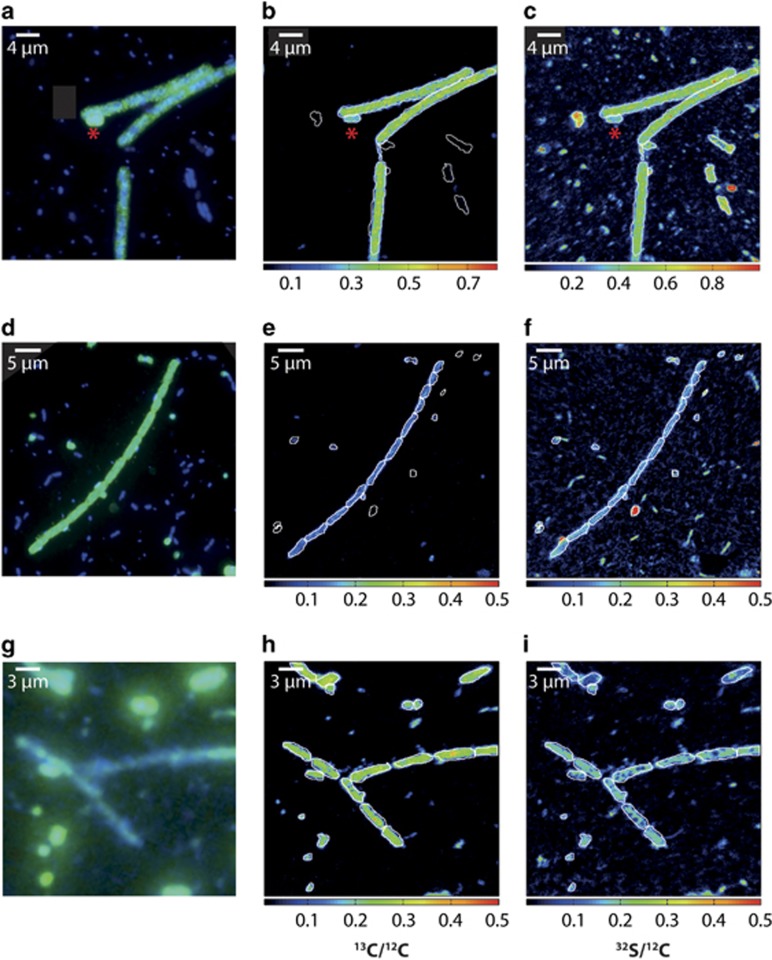
Methane-dependent growth of *Crenothrix* in Lake Rotsee and Lake Zug. (**a**) *Crenothrix* in the Lake Rotsee oxic incubation visualized by CARD-FISH (green; counterstained by DAPI in blue) with a specific probe Creno445 (Stoecker *et al.*, 2006). A small coccoid cell targeted by the probe (marked by the asterisk) might represent a gonidial cell, which *Crenothrix* is reportedly capable of producing (Völker *et al.*, 1977). (**b**) The corresponding ^13^C/^12^C nanoSIMS image shows homogeneous ^13^C enrichment throughout the cell filament. The small coccoid cell is also significantly enriched, albeit less. (**c**) The corresponding ^32^S/^12^C nanoSIMS image showing distribution of organic material on the filter. (**d**) Putative *Crenothrix* filaments in the Lake Zug oxic incubation visualized by DAPI (blue) and CARD-FISH (green) with probe Mgamma669. (**e**) Corresponding ^13^C/^12^C and (**f**) ^32^S/^12^C nanoSIMS images. Note the fragmented nature of the *Crenothrix* filaments and the attached small (unidentified) bacteria. (**g**) Putative *Crenothrix* filaments in the Lake Zug anoxic incubation visualized by DAPI (blue) and CARD-FISH (green) with probe Mgamma669. (**h**) Corresponding ^13^C/^12^C and (**i**) ^32^S/^12^C nanoSIMS images.

Methane oxidation rates were measured in incubations set up in October 2014, with water from the 7 m depth (oxycline), and from 8 m depth (with no detectable oxygen). Water was collected with a Niskin bottle and filled into sterile 1 l Schott bottles without a headspace, closed with butyl stoppers and kept cold and dark until further handling. In the laboratory, 120 ml was distributed into 160 ml serum bottles in an anoxic (N_2_-containing) glove box (Iner Tec, Grenchen, Switzerland), closed with butyl stoppers and crimped. Each incubation was supplemented with ^13^C-labeled methane (99 at%, Campro Scientific, Berlin, Germany) and ^12^C-methane to reach 2  bar overpressure, resulting in ~1.8 mmol l^−1^ CH_4_ in the water phase and 50 at% ^13^C labeling percentage. For comparison, *in situ* methane concentrations at 7 and 8 m depth were ca. 15 and 35 μmol l^−1^ (Supplementary Figure 1). Duplicate bottles were incubated at 6 °C under dark and light conditions along with a control (sterile filtered lake water). Methane oxidation was monitored during an incubation period of 7 days as production of ^13^CO_2_. Anoxically withdrawn water samples (2 ml) were transferred into 6 ml Exetainers (Labco, Lampeter, UK), fixed with 200 μl zinc chloride (50% w/v) and acidified with concentrated H_3_PO_4_ (100 μl). Isotopic ratios of CO_2_ were determined in the headspace with a preparation system (MultiFlow, Isoprime) coupled to an isotope ratio mass spectrometry (Micromass, Isoprime). Subsequently, methane oxidation rates were calculated as described previously (Oswald *et al.*, 2015). These rates are shown in Supplementary Figure 1. As these incubations were unamended (apart from methane addition), aerobic methane oxidation in these incubations was presumably sustained solely by oxygenic photosynthesis (Milucka *et al.*, 2015; Oswald *et al.*, 2015). At selected time points, sub-samples were also taken for catalyzed reporter deposition fluorescence *in situ* hybridization (CARD-FISH) analysis. These data are shown in Supplementary Figure 3. Nanometer-scale secondary ion mass spectrometry (NanoSIMS) and metagenome analyses reported for Lake Rotsee (shown in Figure 1 and Supplementary Figure 2) were performed on samples collected on a previous sampling campaign in August 2013 (rates and other data from this campaign are reported in Oswald *et al.* (2015)).

### Lake Zug nitrate addition experiment

The sampling campaign was carried out in October 2013. Water samples from the anoxic 160 m depth were collected with a Niskin bottle, filled into sterile Schott bottles, closed with a stopper and stored as described above. The water was distributed into sterile 160 ml serum bottles (a 120 ml) in an N_2_ glove box (Mecaplex, Grenchen, Switzerland) as described in detail in Oswald *et al.* (2016). ^13^C-labeled methane (99 at%, Campro Scientific) was supplied at a ~20% labeling percentage. A 2  bar methane overpressure was set using ^12^C-methane. One set of duplicate bottles received no further addition and served as a control and one set of duplicate bottles was amended with ^15^NO_3_^−^ (from a sterile anoxic 100 mmol l^−1^ stock solution) to a final concentration of 50 μmol l^−1^. Bottles were incubated in the dark under *in situ* temperatures (~5 °C) for 16 days. At regular intervals, bottles were subsampled for ^13^CO_2_ measurements in order to determine methane oxidation rates. For this, anoxically withdrawn water samples (2 ml) were transferred into 6 ml Exetainers, fixed with zinc chloride and acidified with concentrated H_3_PO_4_. Isotopic ratios of CO_2_ were determined in the headspace using a Finnigan GasBench II attached to an isotope ratio mass spectrometer (IRMS; Finnigan Delta Plus, Thermo Fisher Scientific, Waltham, MA, USA). Subsequently, methane oxidation rates were calculated as described previously (Oswald *et al.*, 2015). At selected time points, sub-samples were also taken for CARD-FISH and nanoSIMS analyses. An early time point (*T*= 2d) was analyzed by nanoSIMS to obtain data for the calculation of methane uptake rates reported in Table 1. FISH and nanoSIMS images from Lake Zug nitrate incubation (Figure 1; Supplementary Figure 6) originate from the last time point of the incubation (*T*=16 d). The sample for metagenome analysis (sample Z3) was also taken at this time point. Additionally, an *in situ* water sample from 160 m was also used for metagenome analysis (sample Z1). During this sampling campaign, no incubations with added oxygen were performed.

O_2_-supplemented incubations referred to in this manuscript were only performed during a sampling campaign in June 2014 and are described in detail in Oswald *et al.* (2016), where also the corresponding geochemical profiles and methane oxidation rates from relevant depths and incubations are reported. Briefly, O_2_-supplemented incubations were set up as described above, with the difference that instead of nitrate, sterile air was injected to the incubations to reach final O_2_ concentrations of ca. 80 μmol l^−1^ (‘low O_2_’) and ca. 200 μmol l^−1^ (‘high O_2_’), respectively. Incubations were subsampled at regular intervals for methane oxidation rates, CARD-FISH and nanoSIMS analyses. The CARD-FISH and nanoSIMS analyses shown in Figure 1 were performed on samples taken from 160 m incubation after *T*=2 d. The sample for metagenome analysis was taken at the last time point of the ‘low O_2_’ 160 m incubation (*T*=11 d).

### Catalyzed reporter deposition fluorescence *in situ* hybridization

Formaldehyde- (2% (v/v) final concentration) fixed water samples were incubated for 30 min at room temperature before being filtered onto polycarbonate GTTP filters (0.2 μm pore size; Merck Millipore, Darmstadt, Germany). For nanoSIMS analysis, samples were filtered onto Au or Au/Pd-coated GTTP filters (0.2 μm pore size). Permeabilization with lysozyme, peroxidase inactivation, hybridization with specific oligonucleotide probes labeled with horseradish peroxidase in combination with tyramide signal amplification (Oregon Green 488) and DAPI counter staining was performed as described previously (Pernthaler *et al.*, 2002). An overview of probes used (Biomers, Ulm, Germany) is included in Supplementary Table 2. For cell counts and biovolume determinations, one filter was analyzed for each sample. Hybridized filaments (using probe Mgamma669) were enumerated in randomly selected fields of view with a confocal laser scanning microscope (SP5 DMI 6000, Leica, Wetzlar, Germany). For biovolume calculations, length and width of >15 filaments in >10 fields of view were then measured directly in confocal micrographs using LAS AF Lite software (Leica). Values for the cell counts and methane uptake rates of unicellular gamma-MOB cells were taken from Oswald *et al.* (2015) and Oswald *et al.* (2016).

### Nanometer-scale secondary ion mass spectrometry

Areas of interest containing positive CARD-FISH hybridization signals were marked with a laser micro-dissection microscope (DM 6000, Leica Microsystems, Mannheim, Germany). Laser-marked areas were analyzed by nanoSIMS (NanoSIMS 50 l, Cameca, Paris, France) at the MPI Bremen as described previously. For Lake Rotsee (light incubation, 9 m depth), 12 and 26 filaments were analyzed in five fields of view after 2 and 7 days of incubation, respectively. For the Lake Zug low and high O_2_ addition experiments, 19 and 13 filaments were measured in 9 and 7 fields of view, respectively, after 2 days of incubation. For the Lake Zug nitrate addition incubation, 6 filaments were measured in 5 fields of view after 2 days of incubation and 7 filaments were measured in 5 fields of view after 16 days of incubation. Obtained secondary ion images were drift corrected, accumulated and processed with Look@NanoSIMS (Polerecky *et al.*, 2012).

### Biovolume and carbon assimilation rates

The biovolume of individual *Crenothrix* filaments was calculated from their measured length and width by assuming a cylindrical shape. The length and the width of filaments were determined from the CARD-FISH images that were used for cell counting. Due to the varying length of filaments, an average biovolume of *Crenothrix* was calculated and is reported in Table 1. The ‘average biovolume determined from CARD FISH’ was calculated as an average of biovolumes of individual filaments hybridized with a *Crenothrix*-targeting probe (Mgamma669 or Creno445) at the start of the respective incubation and is reported with the s.d. ‘Total’ *Crenothrix* biovolume reported in Table 1 and Supplementary Figure 4 was obtained by multiplying the average filament biovolume by the number of filaments per ml of water. For comparison, the biovolume of unicellular gamma-MOB cells was calculated from total cell counts and by assuming an average spherical cellular diameter of 2 μm.

Cellular ^13^C at % were calculated from ^13^C/^12^C values of individual ROIs (regions of interest). Regions of interests were drawn to outline single *Crenothrix* cells (for example, Figures 1h and i), whole filaments (Figures 1e and f) or parts of filaments (Figures 1b and c). Both the background (cell-free polycarbonate filter in the same field of view) and the ^13^C enrichment of all cells in every field of view was evaluated and compared for all measurements. Rates of methane carbon uptake (fmol C cell_avg_^−1^ d^−1^) of *Crenothrix* and unicellular gamma-MOB were calculated from the ^13^C excess of the measured cells using a conversion factor of 6.4 fmol C μm^−3^ reported in Musat *et al.* (2008). These uptake rates were corrected for the labeling percentage and the incubation time. The methane uptake rates were calculated only for filamentous cells, which were stained with the Creno445 or Mgamma669 probe. Hybridized single cells (such as in Figures 1a–c) were not considered in the calculation.

### DNA extraction, 16S rRNA gene amplicon sequencing and analysis

Two *in situ* water samples from Lake Rotsee were used for 16S rRNA gene amplicon sequencing. One was collected from the oxycline (9 m depth) during a campaign in August 2013 and the other from anoxic water (8 m depth) during a campaign in October 2014 (Supplementary Table 4). Volumes of ca. 250 ml were filtered onto polycarbonate Nuclepore Track-Etched Membrane filters (0.2 μm pore size; Whatman, Maidstone, UK). Filters were stored at −80 °C until DNA was extracted with the UltraClean Soil DNA Isolation Kit (MoBio Laboratories, Carlsbad, CA, USA). Extraction procedure was performed according to manufacturer’s instructions with the following adjustment: vortexing with the Bead Solution was reduced to 30 s with subsequent incubation on ice (30 s), and this cycle was repeated four times.

The V3–V4 regions of the 16S rRNA gene were targeted with primer pair 341 F (5′-CCTACGGGNGGCWGCAG-3′) and 805 R (5′-GACTACCAGGGTATCTAATC-3′). The forward primers contained unique identifier sequences at the 5’-end for each sample to allow for multiplex sequencing. Ten separate PCR reactions (25 μl volume) were set up for each sample including both forward and reverse primers (500 nm each), deoxyribose nucleotide triphosphates (dNTPs; 800 μm), 1 × Taq reaction buffer, Taq DNA polymerase (0.25 U) and DNA extracts of the respective samples (0.5–1 μl). The reactions proceeded as follows: initial denaturation (3 min at 95 °C), 25 cycles of denaturation (30 s at 95 °C), annealing (30 s at 54 °C) and elongation (90 s at 72 °C); and final elongation (10 min at 72 °C). Parallel reactions were combined and purified with the QIA quick PCR Purification Kit (Qiagen, Hilden, Germany) following manufacturer’s instructions, with a final elution in 1 × TE buffer (30 μl; 10 mm Tris-HCl (pH 8.0)+1 mm EDTA). The DNA was further purified with a gel using SYBR Green I Nucleic Acid Gel Stain (Invitrogen, Carlsbad, CA, USA) followed by gel extraction with QIAquick Gel Extraction Kit (Qiagen) according to the manufacturer’s protocol. Extract concentrations were measured fluorometrically using the Qubit dsDNA HS Assay Kit and the Qubit 2.0 Fluorometer (Invitrogen). Illumina sequencing was performed on the amplicons at the Max Planck-Genome Centre (Cologne, Germany).

16S rRNA gene amplicon paired-end reads were trimmed (right end only, trim quality threshold=10) and merged (20 bases minimum overlap) using BBmap software version 35.43 (sourceforge.net/projects/bbmap). Reads were then separated by barcode and trimmed (minimum length=300, maximum homopolymer length=8, maximum number of ambiguous bases=0, minimum average quality score allowed over 50 bp window=20) using mothur v.1.36.1 (Schloss *et al.*, 2009). The separated reads were processed using SILVAngs and standard parameters (Quast *et al.*, 2013).

### Lake metagenome sequencing and assembly

Two *in situ* water samples (Lake Rotsee, 9 m depth, August 2013 (sample R1) and Lake Zug, 160 m depth, October 2013 (sample Z1)) and four end time points of incubations (Lake Rotsee, O_2_-supplemented (sample R2), Lake Rotsee, light (sample R3), Lake Zug, low O_2_-supplemented (sample Z2), Lake Zug, anoxic, nitrate-supplemented (sample Z3); see Supplementary Tables 3 and 4 for additional sample information) were analyzed by Illumina sequencing. The following water volumes were filtered onto polycarbonate Nucleopore Tracked-Etched membrane filters (0.2 μm pore size; Whatman) and stored at −80 °C: 250 ml for *in situ* samples (R1 and Z1), 50 ml for Lake Rotsee incubations (R2 and R3) and 40 ml for Lake Zug incubations (Z2 and Z3). DNA was extracted from cut-up filters using the PowerSoil DNA isolation kit according to manufacturer’s instructions (MoBio Laboratories). DNA from lake Zug was fragmented by sonication (MiSeq: 600–700 bp; HiSeq2500: 300 bp) using a Covaris S2 sonicator (Covaris, Woburn, MA, USA). The library was prepared using Ovation Ultra Low Library Systems V1 (for MiSeq) or V2 (for HiSeq2500) kits (NuGEN Technologies, San Carlos, CA, USA) and paired-end sequencing (2 × 300 or 2 × 150 bp) was performed using the Illumina MiSeq (2 × 300 bp) or HiSeq2500 (2 × 150 bp) platform (Illumina Inc., San Diego, CA, USA). DNA from Lake Rotsee was fragmented by sonication (350 bp) using a Covaris S2 sonicator (Covaris), the library was prepared using NEBNext Ultra DNA Library Prep Kit for Illumina (New England Biolabs, Ipswich, MA, USA) and paired-end sequencing (2 × 150 or 2 × 100 bp) was performed using the Illumina HiSeq2500 or 3000 platform (Illumina Inc.). Both MiSeq and HiSeq sequencing was performed by the Max Planck-Genome-centre, Cologne, Germany (http://mpgc.mpipz.mpg.de/home/; Supplementary Table 3).

Sequences were quality checked using FastQC (Andrews, 2010) and trimming, as well as adapter removal was done using Trimmomatic 0.32 and parameters MINLEN:20 ILLUMINACLIP:TruSeq3-PE.fa:2:30:10 LEADING:3 TRAILING:3 SLIDINGWINDOW:4:15 MINLEN:50 (Bolger *et al.*, 2014). Metagenome assembly of sequences from the Lake Zug incubation (anoxic, nitrate-supplemented (Z3; Supplementary Tables 3, 4)) was performed using SPAdes 3.5.0 (Bankevich *et al.*, 2012) with mismatch corrector enabled and default parameters.

### Sand filter *Crenothrix* metagenome sequencing and assembly

Samples containing high proportions of *C. polyspora* filaments were taken from the backwash water of rapid sand filters of the Wolfenbüttel waterworks (Germany), which treats a mixture of oxic and anoxic groundwater. During sampling, *Crenothrix* filaments were retained from 600 to 850 liters of backwash water by either sedimentation or filtration through a fine-mesh sieve (200 or 400 μm). One sample was collected in 2004 (on 21 June; sample C) and was incubated with 500 μmol l^−1^ ammonium for 212 h. The second sample was collected in 2005 (10 October, sample B) and was incubated at different methane concentrations for 24 h. It should also be noted that earlier we deposited one additional partial and unpublished *Crenothrix* genome from a sand filter sample from the Wolfenbüttel waterworks at IMG (genome ID 3300005627). We did not analyze that older genome sequence in the course of the present study, because it originated from the same site but had been sequenced less deeply than the two sand filter *Crenothrix* genomes described here.

After the incubations, samples B and C were frozen at −20 °C and DNA was extracted in 2016 using a phenol chloroform protocol (Zhou *et al.*, 1996) including two bead-beating steps. Paired-end sample libraries were prepared using Illumina Nextera DNA Library Preparation Kit (Illumina Inc.) and sequenced at Aalborg University (Denmark) using an Illumina MiSeq with MiSeq Reagent Kit v3 (2 × 301 bp; Supplementary Table 3). Paired-end reads were imported to CLC Genomics Workbench v. 8.0 (CLCBio, Aarhus, Denmark) and trimmed using a minimum phred score of 20, a minimum length of 50 bp, allowing no ambiguous nucleotides and trimming off Illumina sequencing adaptors if found. All trimmed paired-end metagenome reads were assembled using CLC’s *de novo* assembly algorithm, using a kmer of 63 and a minimum scaffold length of 1 kbp.

### Metagenome binning, reassembly and annotation

Binning of contigs of the Lake Zug metagenomic assembly (sample Z3, Supplementary Table 3) was performed by exploiting differential contig coverage from three sequenced metagenomic data sets: Z1 (Lake Zug, *in situ*), Z2 (Lake Zug, O_2_-supplemented incubation) and Z3 (Lake Zug, anoxic, nitrate-supplemented incubation) as described previously (Albertsen *et al.*, 2013) and implemented in the mmgenome R package (http://madsalbertsen.github.io/mmgenome/; Karst *et al.*, 2016). Only contigs longer than 500 bp were used and the average coverage of each contig was computed directly using BBmap 35.43 (http://sourceforge.net/projects/bbmap/) with default parameters. Prodigal 2.60 (Hyatt *et al.*, 2010) in metagenomic mode (-p meta) and standard parameters was used to predict open reading frames, which were translated to amino-acid sequences and subsequently searched for using HMMER 3.1b (Eddy *et al.,* 2013) against a set of 107 hidden markov models of essential single-copy genes (Dupont *et al.*, 2012) using default settings and trusted cutoff (-cut_tc) enabled. Protein sequences coding for essential single copy genes were searched against NCBI non-redundant database (retrieved in August 2015) using BLASTP (Camacho *et al.*, 2009) and an e-value cutoff of 10^−6^. The taxonomy (class level) of each essential single-copy gene was assigned using MEGAN5 (Huson *et al.*, 2011; with the previously generated BLASTP xml file as input) and the mmgenome script ‘hmm.majority.vote.pl’. Bowtie2 (Langmead and Salzberg, 2012) with standard settings was used to map reads to contigs and the number of paired-end connections between separate contigs was calculated from the SAM file using the mmgenome script ‘network.pl’.

Differential coverage of contigs between the two sand filter *Crenothrix* metagenomes (Supplementary Figure 8) and between the Lake Zug metagenomes (Supplementary Figure 7), as well as paired-end connections between separate contigs were used to extract genomic bins from the metagenome using the mmgenome R package (http://madsalbertsen.github.io/mmgenome/; Karst *et al.*, 2016). Reads used for the initial assembly were mapped to the binned contigs using BBmap of the BBmap package 35.43 (http://sourceforge.net/projects/bbmap/) using stringent settings (approximate minimum identity=0.98) or CLC (sand filter *Crenothrix*). Mapped reads were reassembled (only for the lacustrine *Crenothrix*) using SPAdes 3.5.0 (Bankevich *et al.*, 2012) with mismatch corrector enabled and default parameters. Quality of the reassembled bins was assessed using CheckM 1.05 running the lineage-specific workflow (Parks *et al.*, 2015). Annotation of the *Crenothrix* D3 draft genome was performed using RAST (Aziz *et al.*, 2008). CDS prediction and automated pre-annotation of the two Wolfenbüttel sand filter *Crenothrix* genome sequence bins were performed using the PROKKA pipeline (Seemann, 2014) with an in-house extended protein reference database. The annotation of key metabolic pathways was manually refined.

The Whole Genome Shotgun project of lacustrine Crenothrix sp. D3 has been deposited at DDBJ/ENA/GenBank under the accession MBQZ00000000. The version described in this paper is version MBQZ01000000. Reads (Lake Zug and Lake Rotsee) have been deposited at the Sequence Read Archive under BioProject PRJNA325574. The two sand filter *Crenothrix* metagenomic assemblies are available in the European Nucleotide Archive (ENA) under the study accession number PRJEB19189.

### Phylogenetic analyses

Full-length amino-acid sequences of bacterial PmoA and AmoA protein sequences were retrieved from the Integrated Microbial Genomes database (IMG-ER; Markowitz *et al.*, 2009) using Pfam family PF02461. Previously published protein sequences of ‘unusual’ PmoA of *C. polyspora* (accession ABC59822–ABC59827; Stoecker *et al.*, 2006), partial PmoA of *C. fusca* (accession ABL64049; Vigliotta *et al.*, 2007) AmoA sequences belonging to Candidatus *Nitrospira nitrosa*, (accession CUS31358; van Kessel *et al.,* 2015) as well as Candidatus *Nitrospira inopinata* (accession CUQ66826; Daims *et al.*, 2015) were added to the reference set. After removing duplicate sequences, protein sequences were aligned using Clustal Omega 1.2.0 (Sievers *et al.*, 2011) and default parameters. A phylogenetic tree (135 taxa) was calculated using RAxML 8.2.6 (Stamatakis, 2014) and parameters: -f a -k -x 48020621 -p 6809427 -N 100 -T 8 -m PROTGAMMAWAG.

Partial *Crenothrix* 16S rRNA gene sequences were retrieved from the *Crenothrix* draft genomes using RNAmmer 1.2 (Lagesen *et al.*, 2007), aligned using the SILVA incremental aligner (SINA) 1.2.11 (Pruesse *et al.*, 2012) and imported to the SILVA SSU NR99_123 database (Quast *et al.*, 2013) using ARB 6.1 (Ludwig *et al.*, 2004). Phylogenetic trees of the 16S rRNA gene sequences were calculated using RAxML 7.7.2 integrated in ARB with the GAMMA model of rate heterogeneity and the GTR substitution model with 100 bootstraps.

## Results and discussion

### *Crenothrix* in Lake Rotsee and Lake Zug

To investigate the potential occurrence of filamentous *Crenothrix* bacteria in two stratified lakes and their involvement in the lacustrine methane cycle, we first recorded geochemical evidence for methane oxidation *in situ*. Concentration profiles recorded in Lake Rotsee and Lake Zug over the course of 3 years suggested a zone of methane consumption that persistently coincided with the oxycline (profiles from Lake Rotsee 2013 are shown in Oswald *et al.* (2015), from 2014 in Supplementary Figure 1; profiles from Lake Zug 2012, 2013 and 2014 are shown in Oswald *et al.*, 2016). Concurrently, incubations with ^13^CH_4_ confirmed high rates of methane oxidation at the oxycline (Oswald *et al.*, 2015, 2016; Supplementary Figure 1). These incubations were set up under both oxic and anoxic conditions. In Lake Rotsee, oxic incubation conditions were obtained either by addition of air or solely by incubation of anoxic water in the light. In the latter case, aerobic methane oxidation was presumably sustained by oxygenic photosynthesis (Milucka *et al.*, 2015; Oswald *et al.*, 2015). In Lake Zug, oxic incubations were solely supplemented with air and incubated in the dark. These different incubation set ups reflected the different nature of the two lakes, Lake Rotsee has a shallow, sun-lit oxycline, whereas the oxycline of Lake Zug is very deep and dark. Additionally, anoxic Lake Zug incubations supplemented with nitrate were also set up as Lake Zug had the appropriate environment to test for methane-dependent denitrification (Supplementary Table 4).

We then analyzed the microbial community at the Lake Rotsee oxycline by 16S rRNA gene amplicon sequencing in 2 consecutive years (2013 and 2014; Supplementary Figure 2). Along with gamma-proteobacterial *Methylococcaceae* (*Methylobacter*, *Methylocaldum*, *Methylomonas* and *Methyloglobulus* species), CABC2E06 (an uncultured *Methylococcales* clone; Wang *et al.*, 2012; Quaiser *et al.*, 2014), and the marine methylotrophic group, also sequences belonging to *Crenothrix* were retrieved. On the basis of the number of recovered sequences, *Crenothrix*-related organisms were 2–5-fold less abundant than *Methylococcaceae* and comprised 0.06–0.1% of the total bacterial sequences *in situ*. However, it is possible that the true abundance of *Crenothrix in situ* was higher than what the 16S rRNA gene abundances suggest, as, for example, DNA extraction biases might strongly select against these thickly sheathed microorganisms.

We could additionally confirm the presence of *Crenothrix* in both lakes by CARD-FISH with two oligonucleotide probes reported to target *Crenothrix*, Mgamma669 and Creno445 (Eller *et al.*, 2001; Stoecker *et al.*, 2006). The more specific oligonucleotide probe Creno445 bound only sporadically, when the hybridization stringency was strongly reduced (Supplementary Figure 3). On the other hand, the Mgamma669 probe hybridized most of the conspicuous filaments in all analyzed samples from both lakes (*in situ* water as well as incubations, Figure 1; Supplementary Figure 3) even though some filaments did not hybridize even with this more general probe (for example, Supplementary Figures 3a, b). With both probes, we observed two hybridized cell morphotypes—filaments and single round cells (Figure 1; Supplementary Figure 3). Both morphotypes have been observed for *Crenothrix* spp. previously and it has been proposed that the smaller round cells represent reproductive cells that bud from the ends of vegetative cell filaments (Cohn, 1870; Völker *et al.*, 1977). However, given the compromised specificity of the Creno445 probe at low stringency and the broad specificity of the Mgamma669 probe, it is also possible that the hybridized single cells represented other gamma-MOB, reportedly targeted by the Mgamma669 probe (for example, *Methylobacter* or *Methylomonas*; Eller *et al.*, 2001). Therefore, the here-reported *Crenothrix* cell counts and biovolumes are solely based on counts of Creno445- or Mgamma669-hybridized filaments and thus represent conservative estimates. Overall, in all analyzed incubations from both lakes total *Crenothix* biovolumes increased over time (Supplementary Figure 4b). This confirms that *Crenothrix* was growing under both oxic and anoxic conditions.

Whereas unicellular gamma-MOB had consistently cell sizes of ca. 2 μm, the individual cells in *Crenothrix*-like filaments reached an average length of ca. 5 μm (Figure 1; Supplementary Figures 3a and 5a). The average length and width of Lake Rotsee *Crenothrix* filaments was ca. 45 and ca. 1.5 μm, respectively, with individual filaments reaching >100 μm length (Supplementary Figure 3). Filaments were often intertwined and bunched together, as observed previously (Cohn, 1870; Völker *et al.*, 1977). In Lake Rotsee, the biovolume of *Crenothrix* was about eight-fold higher than that of unicellular gamma-MOB at depths corresponding to the highest observed methane oxidation rates (in 2012 and 2013; Supplementary Figure 4a). Only in 2014 unicellular gamma-MOB biomass contribution was higher than that of *Crenothrix* (Supplementary Figure 4). We speculate that these differences might be connected to the complex life cycle of *Crenothrix* (Supplementary Discussion). In Lake Zug, the filaments were shorter but more consistent in terms of length, reaching an average length and width of ca. 28 and 1.4 μm (in 2013) and ca. 20 and ca. 1.4 μm (in 2014), respectively.

### Methanotrophic growth of *Crenothrix*

To confirm that the observed cell growth (that is, increase in cell numbers and biovolume over time; Supplementary Figure 4b) was methane-derived, samples from the ^13^CH_4_-supplemented incubations were further analyzed by nanoSIMS. Filamentous bacteria hybridized with the Mgamma669 probe consistently constituted the highest ^13^C-enriched population in all three investigated incubations (Lake Rotsee oxic, Lake Zug oxic and Lake Zug anoxic; Figure 1; Supplementary Figure 5). The ^13^C enrichment confirmed that ^13^CH_4_ was assimilated into cell biomass, such as is common for gamma-proteobacterial methanotrophs (Trotsenko and Murrell, 2008). In some of the images, fragmentation of filaments into single vegetative cells was apparent, even though the uptake of ^13^C appeared homogenously spread throughout the whole filament. In both lakes, *Crenothrix* filaments appeared to be colonized by other non-identified bacteria, which did not show comparably strong enrichment in ^13^C and might thus represent heterotrophic epibionts (Figure 1). In contrast, the single round cells (hybridized with Mgamma669 probe) were similarly enriched in ^13^C as the *Crenothrix* filaments (Figures 1a–c), supporting the speculation that these cells belong to methanotrophic bacteria and might potentially represent reproductive *Crenothrix* cells.

In the Lake Rotsee oxic incubation, the uptake of methane-derived carbon by *Crenothrix* filaments was comparable to that of ‘classical’ unicellular gamma-MOB (^13^C enrichment of 22±4.8 at % and 29±4.1 at %, respectively; Table 1; Figure 1; Supplementary Figure 2). However, due to its larger biovolume *Crenothrix* assimilated ca. 4–6-fold more methane than the ‘classical’ gamma-MOB in the same incubation (1.73 or 1.18 μmol methane l^−1^ d^−1^ and 0.27 μmol methane l^−1^ d^−1^, respectively; Table 1). These numbers are based on average filament biovolumes and cell counts determined by CARD-FISH at the beginning of the incubation and do not take into account any increase in cell numbers over time, as the incubation conditions might have differently affected the growth of the different MOB. However, even if we take into account the increase of cell numbers over time, overall contribution of *Crenothrix* to methane uptake in Lake Rotsee was still higher than that of the unicellular gamma-MOB, even though the difference was not so pronounced (ca. 1.4 higher based on T_end_ cell counts).

*Crenothrix* filaments in Lake Zug oxic incubations were also active and assimilated methane at rates of ca. 0.04 μmol methane l^−1^ d^−1^ (Table 1;  Figures 1d–f). This is much lower than the overall contribution of *Crenothrix* in Lake Rotsee, which is largely due to their lower abundance (1.1E+03 cells per ml) and smaller average biovolume (ca. 30 μm^3^).

Additionally, *Crenothrix* was also active in our anoxic denitrifying incubations where not enough oxygen was present to account for measured methane oxidation rates (2.7 μmol l^−1^ d^−1 13^CO_2_ produced in ^15^NO_3_-supplemented incubation, a ca. 10-fold increase compared to control incubation without any added electron acceptor (0.234 μmol l^−1^ d^−1 13^CO_2_ produced)). The methane-dependent growth under oxygen-deficient conditions was evidenced as cell biomass enrichment in both ^13^C (from ^13^C-CH_4_; Figures 1g–i) and ^15^N (from ^15^N-nitrate; Supplementary Figure 6), even though the methane uptake rates were somewhat lower (0.03 μmol methane l^−1^ d^−1^) than those in incubations supplemented with oxygen (Table 1).

### Metagenomic analyses of Lake Rotsee and Lake Zug

Due to the strong dominance of eukaryotic sequences in Lake Rotsee, we were not able to assemble a genomic bin of *Crenothrix* from any of the sequenced samples (Supplementary Table 1a).

On the other hand, in the Lake Zug metagenomes eukaryotic sequences were almost completely absent and the relative abundance of *Crenothrix*-related sequences was considerably higher (Supplementary Table 1a). Therefore, a metagenome from a Lake Zug anoxic incubation (sample Z3, Supplementary Table 4) was used for the assembly of a *Crenothrix* genome.

**Figure 2:**
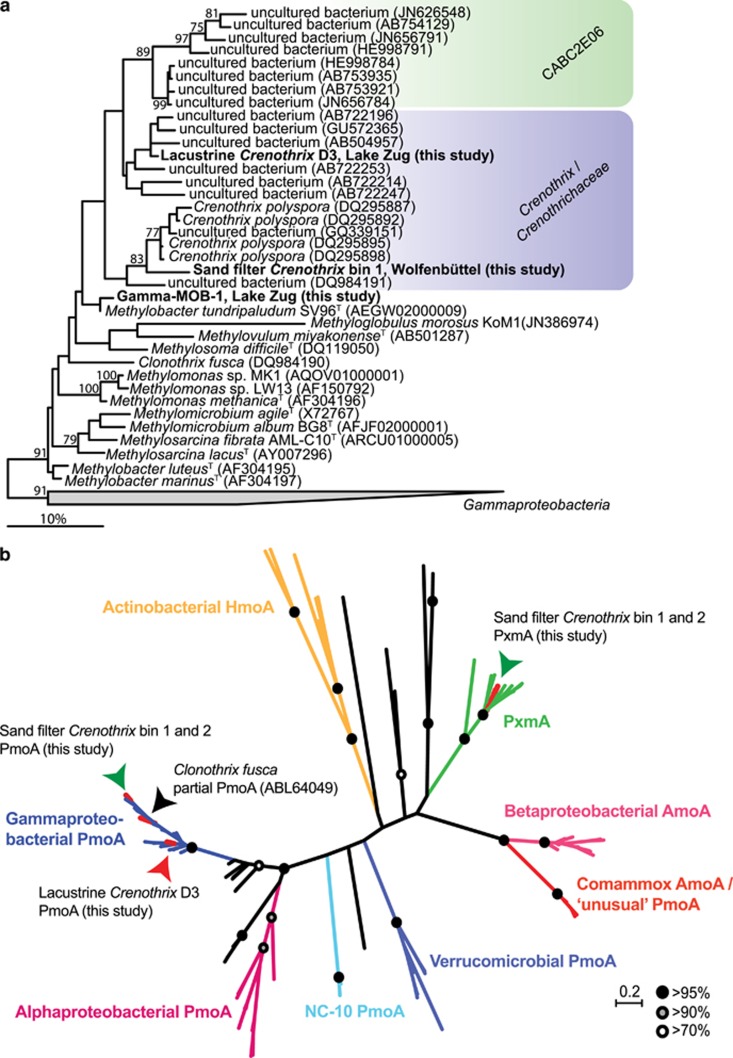
Phylogenetic tree of *Crenothrix* 16S rRNA gene and PmoA amino-acid sequences retrieved from Lake Zug and sand filters of the Wolfenbüttel waterworks. (**a**) Phylogenetic tree of partial 16S rRNA gene sequence retrieved from the lacustrine *Crenothrix* (909 bp) and from one sand filter *Crenothrix* (817 bp, bin 1) draft genomes. Note that the 16S rRNA gene sequence of Lake Zug ‘lacustrine’ *Crenothrix* (but not of the sand filter *Crenothrix*) is monophyletic with clade CABC2E06. The tree was calculated with the RAxML maximum likelihood program implemented in the ARB package without constraining the alignment by a filter or weighting mask. Bootstrap values >70 (out of 100 resamplings) are shown in front of each node. The taxonomic affiliations indicated by the colored boxes are based on the SILVA SSU reference database (release 123; (Pruesse *et al.*, 2007)). Fourteen type strains spread among gamma-proteobacteria were used as an outgroup. Nucleotide accession numbers are listed in brackets. The bar shows an estimated nucleotide sequence divergence of 10%. (**b**) Maximum likelihood phylogenetic tree of bacterial PmoA/AmoA amino-acid sequences (135 taxa) showing affiliation of PmoA sequences recovered from the Lake Zug *Crenothrix* bin (red arrow) as well as of the two sand filter *Crenothrix* genome bins (green arrows). All three *Crenothrix* PmoA sequences clustered within the ‘classical’ gamma-proteobacterial PmoA branch. Bootstrap support of total 100 bootstraps are shown in black (>95%), gray (>90%) and white (>70%) circles. Scale bar indicates substitutions per site.

The *Crenothrix* D3 draft genome was binned by exploiting the differential coverage of contigs in metagenomes obtained from the *in situ* metagenome of Lake Zug and two different incubations (an oxygen-supplemented and an anoxic, nitrate-supplemented; Supplementary Figure 7a; see also Materials and Methods section and Supplementary Table 4 for sample details). We retrieved several bins representing gamma-MOB from the Lake Zug assembly (data not shown). The metagenomic sequences within these two bins were also present in our Lake Rotsee metagenomes, as indicated by their respective coverage (Supplementary Figure 7b). 16S rRNA gene retrieved from one of these bins putatively belonged to a *Methylobacter* (Figure 2a). The other bin contained a partial 16S rRNA gene (909 bp) that clustered closely with *C. polyspora* (Figure 2a), even though the level of similarity (95% identity) suggests that the Lake Zug *Crenothrix* is a different species. Most closely related environmental sequences were retrieved from groundwater and habitats highlighted primarily for iron richness (Bruun *et al.*, 2010), yet apparently containing methane (Kojima *et al.*, 2009; Kato *et al.*, 2013).

Retrieval of the *Crenothrix* D3 16S rRNA gene sequence from the Lake Zug metagenome allowed us to also investigate the reasons behind the poor performance of the Creno445 probe. The comparison of the probe binding region on the 16S rRNA gene sequence revealed that the Creno445 FISH probe (length: 18 nt) had five mismatches with the partial 16S rRNA gene from our metagenomic *Crenothrix* D3 bin (Supplementary Table 2). Interestingly, out of 47 16S rRNA gene sequences in the SILVA database (NR99, release 123) that were assigned to *Crenothrix/Crenothrichaceae,* only seven sequences (including four *C. polyspora* sequences published by Stoecker *et al.* (2006)) contained less than five mismatches. Thus it seems that while the Creno445 probe is very specific to *C. polyspora*, it might not be suitable for environmental detection of other *Crenothrix* strains and species. In comparison, the lacustrine *Crenothrix* 16S rRNA gene had only a single mismatch with the Mgamma669 probe, which explains the comparably better performance of this (not *Crenothrix*-specific) probe on our samples.

Interestingly, the clade CABC2E06, which forms an apparent sister group to *Crenothrix* based on the 16S rRNA tree (Figure 2a), had an identical number of mismatches to both probes. As the 16S rRNA gene sequences assigned to this group were retrieved from both Lake Rotsee (Supplementary Figure 2) and Lake Zug (data not shown), it is feasible that the CABC2E06 bacteria in these samples were also hybridized by the Mgamma669 probe. Additionally, if the CABC2E06 bacteria were filamentous, they may have been included in the here-reported cell and biovolume counts.

### Genome-inferred C1 metabolism of lacustrine *Crenothrix* D3

In the *Crenothrix* D3 draft genome from Lake Zug (Supplementary Table 1b), we searched for pMMO genes. We found all genes encoding for pMMO, which were organized in the arrangement *pmoCAB*, such as is common for gamma-proteobacterial type I MOB (Trotsenko and Murrell, 2008). The phylogenetic analysis of the PmoA amino-acid sequence showed that the sequence fell within the PmoA group of other known gamma-MOB, including the PmoA sequence of the other described filamentous methane oxidizer, *C. fusca* (Figure 2b). However, the presence of conventional gamma-proteobacterial *pmoA* in the lacustrine *Crenothrix* strain was inconsistent with the findings of ‘unusual’ *pmoA* previously reported for *C. polyspora* based on PCR and quantitative PCR (Stoecker *et al.*, 2006). Our *Crenothrix* D3 draft genome did not contain any ‘unusual’ *pmoA;* in fact, no ‘unusual’ *pmoA* or *amoA* has been retrieved in any of the other gamma-MOB-assigned bins either.

We thus decided to address this discrepancy by obtaining metagenomic data from the original samples used in the Stoecker *et al.* (2006) study. Two samples obtained in 2004 from the rapid sand filters of the Wolfenbüttel waterworks (Germany) were analyzed and, after differential coverage binning, genomic information of two *Crenothrix* strains was obtained (Supplementary Figure 8). A partial 16S rRNA sequence retrieved from one sand filter *Crenothrix* bin (bin 1; 817 bp) was 98% identical to the *C. polyspora* 16S rRNA sequence. As the sample reportedly contained high proportions of *C. polyspora*, it is feasible that (at least one of) the sand filter *Crenothrix* was in fact *C. polyspora*. However, throughout this manuscript we refer to these organisms as sand filter *Crenothrix*, without a species name. The sand filter and the lacustrine *Crenothrix* likely represented different species as indicated by the average sequence identities of their shared genes (Supplementary Discussion).

Both genomes of the sand filter *Crenothrix species* contained a *pmoCAB* operon (gene similarities between both bins 96–99%) and a *pxmABC* operon (gene similarities between both bins 93–99%). PmoA encoded by the genes from the *pmoCAB* operon clustered together with other gamma-proteobacterial PmoA sequences (Figure 2b) and the affiliation of the *pxmABC* operon with the sequence-divergent pxm cluster was confirmed by a phylogenetic analysis of *pxmA* (Tavormina *et al.*, 2011; Figure 2b). PxmA has been suggested to play a role in methane oxidation under hypoxic and denitrifying conditions by *Methylomonas denitrificans* and *Methylomicrobium album* (Kits *et al.*, 2015a, 2015b). It thus appears that *Crenothrix* might be another denitrifying methanotroph containing both *pmoCAB* and *pxmABC* operons. Importantly, no ‘unusual’ *pmoA* could be detected in the sand filter *Crenothrix* bins. However, the ‘unusual’ *pmoA* sequence previously assigned to *C. polyspora* was detected in a different bin, clearly belonging to the completely nitrifying *Nitrospira*, apparently co-occurring with *C. polyspora* in the sample (Daims *et al.*, 2015; van Kessel *et al.,* 2015; Pinto *et al.*, 2016). This finding is discussed in more detail in the Supplementary Discussion. It is interesting to note that whereas all three *Crenothrix* PmoA sequences fell within the ‘classical’ gamma-proteobacterial PmoA branch, the lacustrine *Crenothrix* PmoA clustered separate from the sand filter *Crenothrix* bins 1 and 2 and *C. fusca* (Figure 2b). Comparison of the 16S rRNA gene and PmoA amino-acid trees suggested that the PmoA of the lacustrine *Crenothrix* might have been obtained laterally from another gamma-proteobacterial methanotroph. This is supported by the fact that transposase genes were located immediately up- and downstream of the lacustrine *Crenothrix pmoCAB* operon on the respective contig (data not shown).

**Figure 3:**
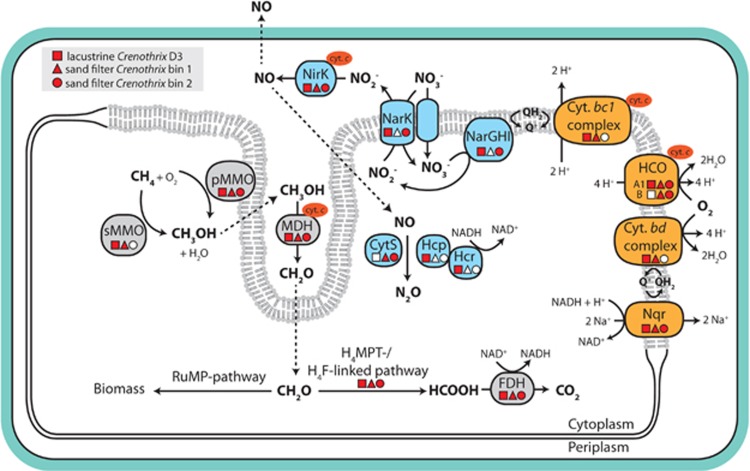
Genome-inferred metabolic potential of *Crenothrix* for respiration and methane oxidation. Predicted metabolic potential of the lacustrine *Crenothrix* as well as of the two sand filter *Crenothrix* species with respect to its CH_4_ and N metabolism inferred from the three draft genomes. Indicated are the methane oxidation pathway (gray boxes), the aerobic respiratory chain (orange boxes) and the pathway for nitrate respiration (blue boxes). Genes that were found in the respective *Crenothrix* genomes (square: lacustrine *Crenothrix* D3; triangle: sand filter *Crenothrix* bin 1; circle: sand filter *Crenothrix* bin 2) are depicted in red, not found in white. Cyt. *bc1* complex, cytochrome *bc1* complex; Cyt. *bd* complex, cytochrome *bd* complex (*cydABCD*); cyt *c*., cytochrome *c*; CytS, cytochrome *c*’-beta; FDH, formate dehydrogenase; H_4_F, tetrahydrofolate; H_4_MPT, tetrahydromethanopterin; HCO, heme copper oxygen reductase (*COXI-III*); Hcp, hybrid cluster protein; Hcr, NADH-dependent Hcp reductase; MDH, methanol dehydrogenase (*xoxF*); Nar, nitrate reductase (*narGHI*); NarK, nitrate/nitrite antiporter (*narK*); NirS, copper-containing nitrite reductase (*nirS*); Nqr, sodium-translocating NADH:quinone oxidoreductase; pMMO, particulate methane monooxygenase (*pmoCAB*); Q, ubiquinone; RuMP, ribulose monophosphate; sMMO, soluble methane monooxygenase (*smmoXYBZDC*).

In addition to the gene cluster encoding for pMMO, we also retrieved a full gene cluster for soluble methane monooxygenase (sMMO; *smmoXYBZDC*) in the lacustrine *Crenothrix* and in one sand filter *Crenothrix* bin. This enzyme is relatively rare in gamma-proteobacterial methanotrophs (Murrell, 2010) and was not found in *C. polyspora* previously (Stoecker *et al.*, 2006), presumably due to the mismatches between the applied PCR primers and the respective target regions in the *mmoX* gene. We cannot conclusively prove involvement of sMMO in methane oxidation by *Crenothrix*; however, as the substrate range of sMMO seems much broader than that of pMMO (Dalton, 2005; Semrau *et al.*, 2011), it is feasible that *Crenothrix* might have the capacity to utilize other C-compounds as suggested previously (Stoecker *et al.*, 2006). This could explain the reported occurrence of *Crenothrix* in, for example, natural bitumen deposits (Saidi-Mehrabad *et al.*, 2013). All three retrieved genomes (two sand filter *Crenothrix* genomes as well as the lacustrine *Crenothrix* D3 genome) further contained all necessary genes for complete oxidation of methane to CO_2_ (Supplementary Discussion; Figure 3).

Like many other type I methanotrophs (Chistoserdova and Lidstrom, 2013), *Crenothrix* might use the RuMP pathway for C1 assimilation from formaldehyde, as genes for all necessary enzymes were found in all three draft genomes (Figure 3). On the other hand, the serine cycle apparently missed genes encoding for hydroxypyruvate reductase and malate thiokinase. *Crenothrix* had the genomic potential for mixed acid fermentation to succinate and potentially acetate (gene encoding for phosphate acetyltransferase was missing in lacustrine *Crenothrix* D3 genome and one sand filter *Crenothrix*, but was putatively present in the other sand filter *Crenothrix* bin) and hydrogen production (via NAD-reducing hydrogenase, *hoxFUYH*; only present in the lacustrine *Crenothrix*). Pyruvate, which serves as the starting point for fermentation, could be generated from formaldehyde via enzymes of the RuMP and pyrophosphate-mediated glycolytic pathway that was encoded in all three *Crenothrix* genomes. Mixed acid fermentation and H_2_ production via these pathways has been shown to be a major route of methane-derived carbon respiration in methanotrophs growing under oxygen limitation (Kalyuzhnaya *et al.*, 2013).

### Aerobic and anaerobic respiration by *Crenothrix*

In agreement with the demonstrated cell growth and activity in our oxic incubations, all three *Crenothrix* genomes encoded a multitude of aerobic respiratory chain complexes, such as a sodium-pumping NADH:ubiquinone oxidoreductase (Na^+^-NQR), the M and L subunits of the NADH:quinone oxidoreductase, the *bc1* complex, an A1-type heme copper cytochrome *c* oxidase, a type B heme copper cytochrome *c* oxidase (only the sand filter *Crenothrix*) and a cytochrome *bd* oxidase that might potentially act as a high-affinity terminal oxidase (Figure 3).

Additionally, the draft genome of the lacustrine *Crenothrix* D3 as well as one of the sand filter *Crenothrix* strains also contained a partial pathway for the respiration of nitrate. We retrieved genes encoding for a membrane-bound respiratory nitrate reductase (*narGHI*), a nitrite/nitrate antiporter (*narK*) as well as a periplasmic multi-copper nitrite reductase (nirK). Genes encoding for nitric oxide (NO) and nitrous oxide (N_2_O) reductases (*norBC* and q-type *nor*, and *nosZ*, respectively) were not found in any of the three bins. Yet, interestingly, all three *Crenothrix* genomes encoded proteins for alternative pathways of NO detoxification to N_2_O. In the genome of *Crenothrix* D3, a gene cluster containing *hcp* and *hcr* genes was found. The *hcp* gene encodes for a unique hybrid cluster protein (Hcp), which has recently been shown to act as a high-affinity NO reductase in *Escherichia coli*, producing N_2_O as the end product (Wang *et al.*, 2016). The Hcp sequence retrieved from the *Crenothrix* D3 genome contained the six highly conserved residues involved in 4Fe-2S-2O cluster coordination (Aragao *et al.*, 2008) as well as a glutamic acid residue (E492 of E*. coli* Hcp) essential for NO reductase activity (Wang *et al.*, 2016). Overall, the *Crenothrix* D3 Hcp shared 49% amino-acid identity with the NO-reducing Hcp of E. *coli*. The *hcr* gene, located immediately downstream from *hcp*, encodes for the Hcr protein and acts as a NADH-dependent Hcp reductase (van den Berg *et al.,* 2000), while simultaneously protecting Hcp from nitrosylation by its substrate, NO (Wang *et al.*, 2016). The *hcp/hcr* genes in *Crenothrix* D3 genome were preceded by *norR*, a transcriptional regulator of three different enzymes (NO reductase, flavorubredoxin and flavohaemoglobin) that all utilize NO as a substrate (Rodionov *et al.*, 2005). We thus speculate that, despite being routinely annotated as a hydroxylamine reductase, the Hcp/Hcr system in *Crenothrix* could in fact act as a NO reductase and substitute Nor-type NO reductases under denitrifying conditions. In the two sand filter *Crenothrix* genome bins no homologs of Hcp were found. However, both bins (but not the lacustrine *Crenothrix* genome bin) contained a homolog of cytochrome *c*'-beta, a member of the cytochrome P460 family found in, for example, gamma-proteobacterial methane oxidizers (Zahn *et al.*, 1996; Campbell *et al.*, 2011) and gamma- and beta-proteobacterial ammonia oxidizers (Bergmann and Hooper, 2003; Klotz *et al.,* 2006). Cytochrome *c*'-beta can reduce NO to N_2_O (Elmore *et al.*, 2007). Interestingly, in one of the bins this gene (*cytS*) was located directly downstream of the *haoA* and *haoB* genes encoding for hydroxylamine dehydrogenase. As both the Hcp and the cytochrome *c*’-beta are predicted to be cytoplasmic proteins and NO is produced in the periplasm (by NirK), it is feasible that their activities are not coupled and (some) NO might escape out of the cell (Figure 3).

The experimentally demonstrated and genome analysis-supported metabolic potential for methane-dependent growth under nitrate-reducing conditions cannot serve as a final proof of nitrate reduction by *Crenothrix* in Lake Zug. However, it is interesting to speculate that such metabolic versatility might expand the habitat of these facultative anaerobic bacteria, potentially enabling them to survive periods of oxygen starvation by switching to using nitrate as an electron acceptor for methane oxidation. Denitrification is an emerging feature of gamma-MOB, which has been supported by genomics and was also experimentally demonstrated (Hoefman *et al.*, 2014; Kalyuzhnaya *et al.*, 2015; Skennerton *et al.*, 2015; Kits *et al.*, 2015a, 2015b). It has been proposed that respiration of nitrate might enable aerobic gamma-MOB to colonize anoxic waters (Chistoserdova, 2015; Knief, 2015). In Lake Rotsee and Lake Zug, *Crenothrix* was indeed found in the anoxic waters below the oxycline in at least 2 consecutive years. Its abundance in anoxic lake waters suggests that it might successfully compete with more obligate anaerobic methane oxidizers, such as archaeal methanotrophs (Haroon *et al.*, 2013) or ‘*Candidatus* Methylomirabilis oxyfera’ (Ettwig *et al.*, 2010).

## Conclusions

Members of the genus *Crenothrix* are rare methane oxidizers, which are not available in pure or enrichment cultures and will not be readily picked up in environmental samples by the currently available specific FISH probe (Creno445). The ambiguity surrounding their *pmoA* has further complicated the *in situ* detection using molecular methods. In the past, this has hampered our understanding of these peculiar organisms and possibly led us to underestimate their role in the biogeochemical nutrient and element cycles.

In our study, we could unambiguously demonstrate a key role for these organisms in the mitigation of methane emissions from two stratified lakes. In Lake Rotsee, *Crenothrix* even contributed more to methane uptake than the ‘classical’ unicellular gamma-MOB. In up to 3 consecutive years *Crenothrix* was recurrently found throughout the stratification period of Lake Rotsee and Lake Zug, and thus appears to be a stable part of the indigenous microbial community. Our data are also the first to demonstrate that *Crenothrix* is capable of growing as a planktonic species in the lake water column. Given the capacity of *Crenothrix* to rapidly grow up into large biomass, its participation in methane cycling also in other relevant habitats should be considered.

## Figures and Tables

**Table 1:** Overview of methane carbon uptake rates by *Crenothrix* and unicellular gamma-MOB in Lake Rotsee and Lake Zug

	^*13*^*C at %*^a^ *(*n*)*	*Avg biovolume (CARD-FISH-based)*^b^ *(μm*^*3*^*;*n*)*	*Methane-C uptake per cell*^c^ *(fmol cell*_*avg*_^−*1*^ *d*^−*1*^)	*Cell count*^d^ *(cell ml*^−*1*^)	*Total population biovolume*^e^*(μm*^*3*^ *ml*^−*1*^)	*Methane-C uptake per population*^f^ *(μmol l*^−*1*^ *d*^−*1*^)
*Lake Rotsee (oxic)*						
*Crenothrix* (Mgamma669)	NA	85±8.3 (59)	147.7±26.3^g^	1.2E+04	1.0E+06	1.73^g^
*Crenothrix* (Creno445)	22.00±4.8 (17)	73.7±8.4 (51)	128.0±22.8	9.2E+03	6.8E+05	1.18
Other gamma-MOB^h^	28.77±4.1	4.2	10.6±0.9	2.6E+04	1.1E+05	0.27
						
*Lake Zug (oxic)*						
*Crenothrix* (Mgamma669; low O_2_)	9.26±1.7 (19)	32.5±5.5 (20)	38.1±6.9	1.1E+03	3.5E+04	0.041
*Crenothrix* (Mgamma669; high O_2_)	8.68±1.9 (10)^i^	32.5±5.5 (20)	35.3±7.8	1.1E+03	3.5E+04	0.038
Other gamma-MOB (low O_2_)^j^	10.39±3.1	4.2	5.7±1.2	6.8E+04	2.9E+05	0.39
Other gamma-MOB (high O_2_)^j^	12.13±3.75	4.2	6.9±1.6	6.8E+04	2.9E+05	0.47
						
*Lake Zug (anoxic)*						
*Crenothrix* (Mgamma669)	13.27±4.9 (6)	49.7±20.3 (15)	74.2±26.6 (6)	0.4E+03	2.0E+04	0.03
Other gamma-MOB	NA	NA	NA	NA	NA	NA

Abbreviations: CARD-FISH, catalyzed reporter deposition fluorescence *in situ* hybridization; MOB,

methane-oxidizing bacteria; n, number of analyzed cells, NA, not analyzed.

a Calculated as an average (± s.d.) of the ^13^C/^12^C ratios of individual regions of interest (i.e., cells) determined by nanoSIMS.

b Calculated from CARD-FISH data as an average biovolume (± s.d.) using Mgamma669 or Creno445 probe (*Crenothrix*) and Mgamma84+705 probes (other gamma-MOB).

c Calculated as follows: data from column a were converted into ^13^C excess in fmol per cell (of a given average biovolume; cell_avg_) using the avg cell biovolume reported in column b and a conversion factor of 6.4 fmol C μm^−3^ (Musat *et al.*, 2008). The numbers were corrected for labeling percentage and incubation time.

d Counted from the same filters from which avg biovolumes (column b) were obtained. As the boundaries between individual cells within the filament were often not recognizable, only hybridized filaments were counted. Cell counts refer to cell abundances at the start of each incubation and thus do not account for increase of cell abundances during the incubation period.

e Calculated as follows: data from column b were upscaled using data in column d.

f Calculated as follows: data from column c were upscaled using data from column d.

g Assuming the same ^13^C enrichment as determined with the probe Creno445 on the same sample.

h According to Oswald *et al.*, 2015.

i In this sample, three analyzed filaments had ^13^C/^12^C<0.015 and were not included in the analysis.

j According to Oswald *et al.*, 2016.

Calculations are based on incubations from Lake Rotsee (oxic, 2013) and Lake Zug (oxic and anoxic, 2013, 2014; see Supplementary Table 4 for sample details).
