# Supplementary material for: Crenothrix are major methane consumers in stratified lakes
Source: ISME J. 2017 Jun 6;11(9):2124–40. doi: 10.1038/ismej.2017.77 (PMC5563964; doi:10.1038/ismej.2017.77)
Supplement: Supplementary Information [file ismej201777x1.docx]

***Supplementary Information File***

***Crenothrix* are major methane consumers in stratified lakes**

Kirsten Oswald, Jon S. Graf, Sten Littmann, Daniela Tienken, Andreas Brand, Bernhard Wehrli, Mads Albertsen, Holger Daims, Michael Wagner, Marcel M.M. Kuypers, Carsten J. Schubert & Jana Milucka

**Supplementary Discussion**

***Life cycle of* Crenothrix *and lake turnover***

In Lake Rotsee, the proportion of small gonidial to large filamentous cells varied between sampling years. In 2012 and 2013 we observed many long intact filaments (Supplementary Figure 3), whereas in 2014 we only detected short *Crenothrix* fragments and gonidial cells seemed to be more numerous. In 2012 and 2013 our sampling was conducted in August, when stratification was stable and methane fluxes had probably reached their maximum (13±3 mmol m^-2^ d^-1^; Oswald *et al.,* 2015) whereas in 2014 the sampling campaign was conducted in late October and though the lake still showed stable stratification below 7 m depth, the methane fluxes were somewhat lower (8±2 mmol m^-2^ d^-1^; Supplementary Figure 1). It is possible that filaments may have propagated just before the lake overturn which increased the ratio of comparably small gonidial cells to long vegetative cells. These changes in their life cycle could explain the comparably lower *Crenothrix* biovolume contribution in 2014.

***Average nucleotide and amino acid identities of lacustrine and sand filter* Crenothrix**

The average nuceotide identities (ANI; Richter and Rosselló-Móra 2009) between lacustrine *Crenothrix* D3 and the two sand filter *Crenothrix* genomes were 72.9 - 73% and 80.5 % between the latter two genomes, respectively. These values are far below proposed species delineation boundaries of 95 - 97% (Goris *et al.,* 2007, Varghese *et al.,* 2015). The average amino acid identity (AAI; Konstantinidis and Tiedje 2005) between lacustrine *Crenothrix* D3 and the two sand filter *Crenothrix* species is 66.1 – 66.4 % and the AAI between the two latter genomes is 77.4%. These AAI values are above the proposed genus delineation boundary of 60% (Luo *et al.,* 2014) suggesting that all three *Crenothrix* species indeed belong to the same genus. It should be noted that also other members of the *Methylococcaceae* (such as *Methyloglobulus morosus*; Deutzmann *et al.,* 2014) have AAI values with the three *Crenothrix* genomes that suggest affiliation to the same genus (data not shown), and therefore the taxonomy of this order might need to be revised.

***Extended genome description of sand filter* Crenothrix *species and the lacustrine* Crenothrix D3**

*Downstream oxidation of methanol*

All *Crenothrix* genomes contained a *XoxF* homolog encoding for the large subunit of the pyrroloquinoline quinone- and cerium-dependent methanol dehydrogenase (MDH*)*, an enzyme catalyzing downstream oxidation of methanol to formaldehyde or formate. Interestingly, *mxa* genes encoding for the calcium-dependent MDH and its accessory proteins were not found in the lacustrine *Crenothrix* D3 draft genome and sand filter *Crenothrix* bin 2 but were found in sand filter *Crenothrix* bin 1. Absence of *mxa*-type MDHs in genomes containing *xoxF*-type MDHs have so far been described for several methylotrophs (Chistoserdova 2011, Giovannoni *et al.,* 2008, Kalyuhznaya *et al.,* 2009, Wilson *et al.,* 2008) as well as verrucomicrobial methanotrophs (Khadem *et al.,* 2012, Op den Camp *et al.,* 2009, Pol *et al.,* 2014). Genes encoding for enzymes catalyzing a four-step C1 interconversion of formaldehyde to formate via the methenyl-tetrahydromethanopterin pathway (*fae*, *mtdB*, *mch*, *fhc*) were all present, in both the lacustrine and the sand filter *Crenothrix* genomes. The alternative tetrahydrofolate (H_4_F)-linked pathway was missing FolD, the bifunctional enzyme acting as methylene-H_4_F dehydrogenase and methenyl-H_4_F cyclohydrolase. However, in the case of *Crenothrix*, this enzyme might be substituted by Fch and Mtd, such as has been shown for other methylotrophs (Chistoserdova 2011). These genes (*fch, mtdB*, and several *mtd* homologues) were found in the *Crenothrix* genomes. In the last step, formate can further be oxidized to CO_2_ by a NAD-dependent formate dehydrogenase, which was encoded in all three *Crenothrix* draft genomes.

*Carbohydrate metabolism (only annotated for the lacustrine Crenothrix)*

All genes encoding for core enzymes involved in the pentose phosphate pathway, tricarboxylic acid cycle, Entner-Doudoroff pathway as well as Embden-Meyerhof-Parnas pathway were present in the lacustrine *Crenothrix* D3 draft genome.

*Nitrogen assimilation (only annotated for the lacustrine Crenothrix)*

Genes encoding for assimilatory nitrate and NAD(P)H-dependent nitrite reductase were retrieved from the lacustrine *Crenothrix* D3 draft genome. Downstream assimilation of ammonium can proceed via the GS/GOGAT-pathway by glutamine synthetase and glutamate synthase which genes were both present in the genome.

*Nitrogen fixation*

Lacustrine *Crenothrix* might also have the potential to fix dinitrogen gas since the genome contained key genes encoding for nitrogenase as well as a suite of its accessory proteins (i.e. *nifKDHWENX*). Nitrogenase genes were absent from both sand filter *Crenothrix* draft genomes, with the exception of *nifK* in bin 1.

***Discussion of the canonical gamma-proteobacterial and ‘unusual’* pmoA *sequence in the sand filter* Crenothrix**

Stoecker *et al.* (2006) have retrieved ‘unusual’ *pmoA* from a sample strongly dominated by filaments that were identified by morphology as *C. polyspora*. *C. polyspora* abundance was observed by FISH using a *Crenothrix*-specific probe as well as Bacteria- and Archaea- and Eukarya-specific FISH probes and was independently confirmed by qPCR using two general and two *C. polyspora*-specific 16S rRNA gene targeting primer sets [Figure 3 of the Stoecker *et al.* (2006) paper]. By using additional qPCR assays for the ‘unusual’ *pmoA* (two assays) and canonical gamma-proteobacterial *pmoA* (two assays) a much higher abundance of the ‘unusual’ than the canonical *pmoA* was observed and thus it was concluded that *C. polyspora* very likely encodes the ‘unusual” *pmoA* gene. This conclusion was further supported by the fact that transcription of the ‘unusual’ *pmoA* was strongly induced by methane addition [Figure 7 in the Stoecker *et al.* (2006) paper]. Surprisingly, we and others recently demonstrated that completely nitrifying *Nitrospira* species (comammox) encode *amoA* genes that are highly similar to the ‘unusual” *pmoA* genes assigned to *C. polyspora* (Daims et al., 2015, Palomo et al., 2016, Pinto et al., 2016, van Kessel et al., 2015).

To address this issue, we obtained frozen material from the sample used in the Stoecker *et al.* (2006) paper (this sample material had been used for unpublished incubation experiments before freezing) and reconstructed two draft *C. polyspora* genomes by metagenomic sequencing. Interestingly, these genomes had ANI values that demonstrated that they represent two different *Crenothrix* species to which we thus refer to as sand filter *Crenothrix* species in this manuscript. In both genome bins the canonical gamma-proteobacterial methane monooxygenase (in addition to another gamma-proteobacterial *pmoABC* operon; see main text) was encoded, while the ‘unusual’ *pmoA* could not be detected. However, the ‘unusual’ pmoA previously assigned to *C. polyspora* was detected in the metagenome from this sample in one of the two comammox *Nitrospira* bins (see Supplementary Figure 8; the other comammox *Nitrospira* bin contains another ‘unusual’ *amoA*). We thus conclude from these data that in the sample used by Stoecker *et al.* 2006 comammox *Nitrospira* thrived, which encode the ‘unusual’ *pmoA* (and use it as *amoA*), and that the two *C. polyspora* strains encode the canonical gamma-proteobacterial *pmoA* [which was also retrieved in the Stoecker et al. (2006) study but assigned to another gamma-MOB in the sample, *Methylomicrobium album*]. We do not have a conclusive explanation for the qPCR data shown in Figure 3 in the Stoecker *et al.* (2006). The strongly increased transcription of the ’unusual’ *pmoA* gene after addition of methane to the *C. polyspora*-dominated sample as described by Stoecker *et al.* could either be explained by the existence of a low abundant methane-oxidizing *Crenothrix* strain possessing this gene (as speculated below) or by methane-induced secretion of metabolites by *C. polyspora* that stimulated the comammox *Nitrospira* in the sample. In the Stoecker *et al.* (2006) study DNA for the qPCR assays was extracted using the FastDNA kit (QBiogene, Irvine, CA) while the much harsher phenol chloroform bead beating protocol was used in our metagenome analysis of the same sample. One could thus speculate that the FastDNA kit did not lyse the dominant populations of *Crenothrix* strains (with canonical gamma-proteobacterial *pmoA*) and only DNA from a low abundant *Crenothrix* strain (not binned in the metagenome) was obtained, whose canonical *pmoA* has been replaced by the comammox *amoA*. The existence of comammox *Nitrospira* that are very closely related to purely nitrite-oxidizing *Nitrospira* strains, which do not possess the ’unusual’ *pmoA*/*amoA* gene, indicate lateral gene transfer events of the genes necessary for ammonia oxidation, and suggest a complex evolutionary history of these genes (Daims *et al.,* 2015).

**Supplementary Table 1a. Average coverage of the lacustrine *Crenothrix* D3 draft genome in all metagenomic data sets of Lake Zug and Lake Rotsee.** See Supplementary Table 3 for additional information on the metagenomic data sets.

|  | **Lake Zug (metagenomic data sets Z1-3)** | | | **Lake Rotsee (metagenomic data sets R1-3)** | | |
| --- | --- | --- | --- | --- | --- | --- |
|  | ***in situ* (Z1)** | **oxic (Z2)** | **anoxic (Z3)** | ***in situ* (R1)** | **oxic (O_2-_supplemented; R2)** | **oxic (light; R3)** |
| Lacustrine *Crenothrix* D3 | 2.2 | 16.8 | 23.1 | 0.01 | 0.3 | 0.13 |

**Supplementary Table 1b. Summary statistics of sand filter *Crenothrix* and lacustrine *Crenothrix* D3 draft genomes.** N50 is a length weighted median of contig length and is defined as the shortest contig length needed to cover 50% of the metagenomic bin. Completeness, contamination and strain heterogeneity were assessed by CheckM (Parks *et al.,* 2015) using 290 lineage-specific marker sets of Gamma-proteobacteria.

| **Metagenomic bin** | **Length (Mb)** | **GC content** | **No. of contigs (N50)** | **Completeness/Contamination/Strain heterogeneity (%)** |
| --- | --- | --- | --- | --- |
|  |  |  |  |  |
| Lacustrine *Crenothrix* D3 | 3.63 | 42.2% | 103 (89.8 Kb) | 98.4/ 1.8/ 0.0 |
| Sand filter *Crenothrix* bin 1 | 3.97 | 44.2% | 180 (51.2 Kb) | 98.3/ 1.4/ 0.0 |
| Sand filter *Crenothrix* bin 2 | 3.57 | 44.7% | 467 (11.7 Kb) | 92.1/ 3.6/ 57.1 |

**Supplementary Table 2. Overview of used oligonucleotide probes**

Listed are target groups, 5’-3’ sequence, % [v/v] formamide in the hybridization buffer and respective references.

| **Probe** | **Target group** | **Probe sequence**  **(5'- 3')** | **% Formamide** | **Mismatch (nt)*** | **Reference** |
| --- | --- | --- | --- | --- | --- |
| Creno445 | *Crenothrix polyspora* | GCT TGC CTT TTT CCT CCC | 0-35^§^ | 5 | Stoecker *et al.,* 2006 |
| EUB338 I-III | most bacteria | GCT GCC TCC CGT AGG AGT  GCA GCC ACC CGT AGG TGT  GCT GCC ACC CGT AGG TGT | 35 | 0 (EUB338 I)  3 (EUB338 II)  2 (EUB338 III) | Daims *et al.,* 1999 |
| Mgamma84 | type-I methanotrophs | CCA CTC GTC AGC GCC CGA | 20 | 2 | Eller *et al.,* 2001 |
| Mgamma705 |  | CTG GTG TTC CTT CAG ATC |  | 1 |  |
| Mgamma669 | *Crenothrix, Methylobacter, Methylomonas* | GCT ACA CCT GAA ATT CCA CTC | 20 | 1 | Eller *et al.,* 2001 |

*Mismatch of the retrieved lacustrine *Crenothrix* D3 16S rRNA gene sequence with the respective probe (number of nucleotides)

^§^For Creno445 probe various formamide concentrations were used

**Supplementary Table 3. Summary of raw metagenomic sequences obtained from Wolfenbüttel waterworks sand filters, Lake Zug and Lake Rotsee (*in situ* and *in vitro* incubations).** See Supplementary Table 4 for additional information on the origin of sequenced samples.

| **Metagenome sample [identifier]** | **Sequencing technology** | **No. of paired-end reads** | **Total sequenced (Gb)** | **Sample origin and date** |
| --- | --- | --- | --- | --- |
| Lake Zug, *in situ* [Z1] | MiSeq (2x300bp) | 7,401,029 | 4.4 | Lake Zug water column (160 m), *in situ*, October 2013 |
| Lake Zug, oxic [Z2] | HiSeq2500 (2x100bp) | 44,545,098 | 8.9 | Lake Zug, water column (160 m), O_2_-supplemented dark incubation (t=11 d), June 2014 |
| Lake Zug, anoxic [Z3] | MiSeq (2x300bp) | 8,766,855 | 5.3 | Lake Zug, water column (160 m), nitrate-supplemented dark incubation (t=16 d), October 2013 |
| Lake Rotsee, *in situ* [R1] | HiSeq2500 (2x100bp) | 45,352,147 | 9.1 | Lake Rotsee, water column (9 m), *in situ*, August 2013 |
| Lake Rotsee, oxic (O_2_-supplemented) [R2] | HiSeq3000 (2x150bp) | 81,632,757 | 24.5 | Lake Rotsee, water column (9 m), O_2_-supplemented dark incubation (t=11 d), August 2013 |
| Lake Rotsee, oxic (light) [R3] | HiSeq2500 (2x100bp) | 43,258,628 | 8.7 | Lake Rotsee, water column (9 m), light incubation (t=11 d), August 2013 |
| Wolfenbüttel sand filter *Crenothrix*, sample B | MiSeq (2x301 bp) | 1,841,331 | 1.1 | Wolfenbüttel waterworks, sand filter, October 2005 |
| Wolfenbüttel sand filter *Crenothrix*, sample C | MiSeq (2x301 bp) | 2,406,567 | 1.4 | Wolfenbüttel waterworks, sand filter, June 2004 |

**Supplementary Table 4. Overview of samples from Lake Rotsee, Lake Zug and Wolfenbüttel waterworks (rapid sand filters) analyzed and shown in this study.** Indicated are sample treatment, performed analyses and relevant figures. For detailed description of incubation experiments see Materials and Methods section in this manuscript and Oswald *et al*., 2015, 2016, respectively. Additional information on the metagenomic datasets R1-3, Z1-3, and B and C can be found in Supplementary Table 3.

| **relevant samples*** | | **detailed description** | **methane oxidation rates** | **FISH,**  **cell counts,**  **biovolumes** | **nanoSIMS** | **16S rRNA**  **amplicon sequencing** | **metagenome sequencing** |
| --- | --- | --- | --- | --- | --- | --- | --- |
| **Lake Rotsee 2013** | | water sample from the oxycline, sampled in August |  |  |  |  |  |
| *in situ* | | 9 m | Oswald *et al.,* 2015 | this study  (SupplFig 3, 4) |  | this study  (SupplFig 2) | sample R1 |
| oxic (light) | | T=2d (FISH, nanoSIMS);  T_end_=11d (metagenome) | Oswald *et al.,* 2015 | this study  (Table 1; Fig 1; SupplFig 3, 4, 5) | this study  (Table 1; Fig 1; SupplFig 5) |  | sample R3 |
| oxic (O_2_-supplemented) | | T_end_=11d | Oswald *et al.,* 2015 |  |  |  | sample R2 (SupplFig 7) |
| **Lake Rotsee 2014** | | water sample from the oxycline, sampled in October |  |  |  |  |  |
| *in situ* | | 7 m | this study (SupplFig1) | this study  (SupplFig 4) |  |  |  |
|  |  | 8 m | this study (SupplFig1) | this study  (SupplFig 3) |  | this study  (SupplFig 2) |  |
|  | |  |  |  |  |  |  |
| **Lake Zug 2013** | | water sample from an anoxic depth, sampled in October |  |  |  |  |  |
| *in situ* | | 160 m |  | this study  (SupplFig 4) |  |  | sample Z1 (SupplFig 7) |
| anoxic (NO_3_-supplemented) | | 50 μmol l^-1 15^NO_3_^-^;  T=2d (nanoSIMS); T_end_=16 d (FISH, nanoSIMS, metagenome) |  | this study  (Table 1; Fig 1, SupplFig 4) | this study  (Table 1; Fig 1, SupplFig 6) |  | sample Z3 (SupplFig 7) |
| **Lake Zug 2014** | | water sample from an anoxic depth, sampled in June |  |  |  |  |  |
| *in situ* | | 160 m | Oswald *et al.,* 2016 | this study  (SupplFig 4) |  |  |  |
| oxic (O_2_-supplemented;  low O_2_) | | 80 μmol l^-1^ O_2_; T=2 d (FISH, nanoSIMS); T_end_=11 d (metagenome) | Oswald *et al.,* 2016 | this study  (Table 1; Fig1, Suppl Fig 4) | this study  (Table 1) |  | sample Z2 (SupplFig 7) |
| oxic (O_2_-supplemented;  high O_2_) | | 200 μmol l^-1^ O_2_; T=2 d (FISH, nanoSIMS); T_end_=11 d (metagenome) | Oswald *et al.,* 2016 | this study  (Table 1; Fig1, Suppl Fig 4) | this study  (Table 1; Fig1) |  |  |
| **Wolfenbüttel waterworks Sample C (21.6.2004)** | | Sieved backwash water of rapid sand filters. Sample was incubated for 190 to 240 h at 4 to 40°C with 3 µmol l^-1^ dissolved methane and subsequently stored at -20°C until DNA extraction. |  |  |  |  | Sample C  (SupplFig 8) |
| **Wolfenbüttel waterworks Sample B (18.10.2005)** | | Sieved backwash water of rapid sand filters. Sample was incubated for 24h at 20°C with 0.15 to 125 µmol l^-1^ dissolved methane and subsequently stored at -20°C until DNA extraction. |  |  |  |  | Sample B (SupplFig 8) |
| *all incubations were supplemented with methane in excess | | |  |  |  |  |  |
|  |  | |  |  |  |  |  |
|  |  | |  |  |  |  |  |

**
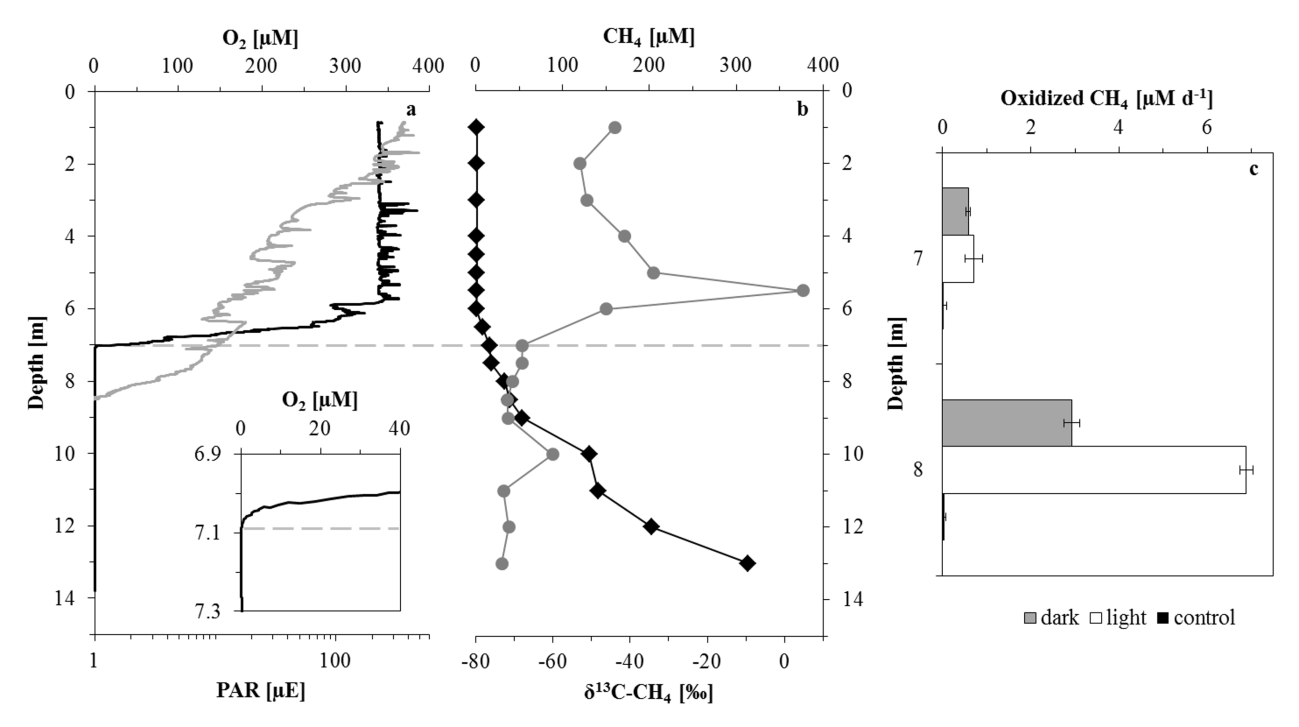
**

**Supplementary Figure 1. Hydrochemical conditions and methane oxidation rates in Lake Rotsee in October 2014.**

**a,** Depth profiles of oxygen (black line) and photosynthetically active radiation (PAR; grey line). The inset shows the exact location of the oxycline at 7.1 m measured with the trace oxygen optode. Note the logarithmic scale for PAR and that light penetrated below the oxycline (dashed line). **b,** Methane concentrations (black diamonds) and corresponding stable carbon isotopes of methane (grey circles). The isotopic signature of methane became substantially heavier at and above the oxycline, indicative of biological methane oxidation. The sharp peak in the methane isotopic ratio profile at ca. 5.5 m depth is indicative of a local source supplying methane at this depth (by *in situ* production or by lateral transport from littoral sediments). **c,** methane oxidation rates under dark and light conditions with water from the oxycline (7 m) and the anoxic waters below (8 m). No methane oxidation was detected in a sterile-filtered control.

**
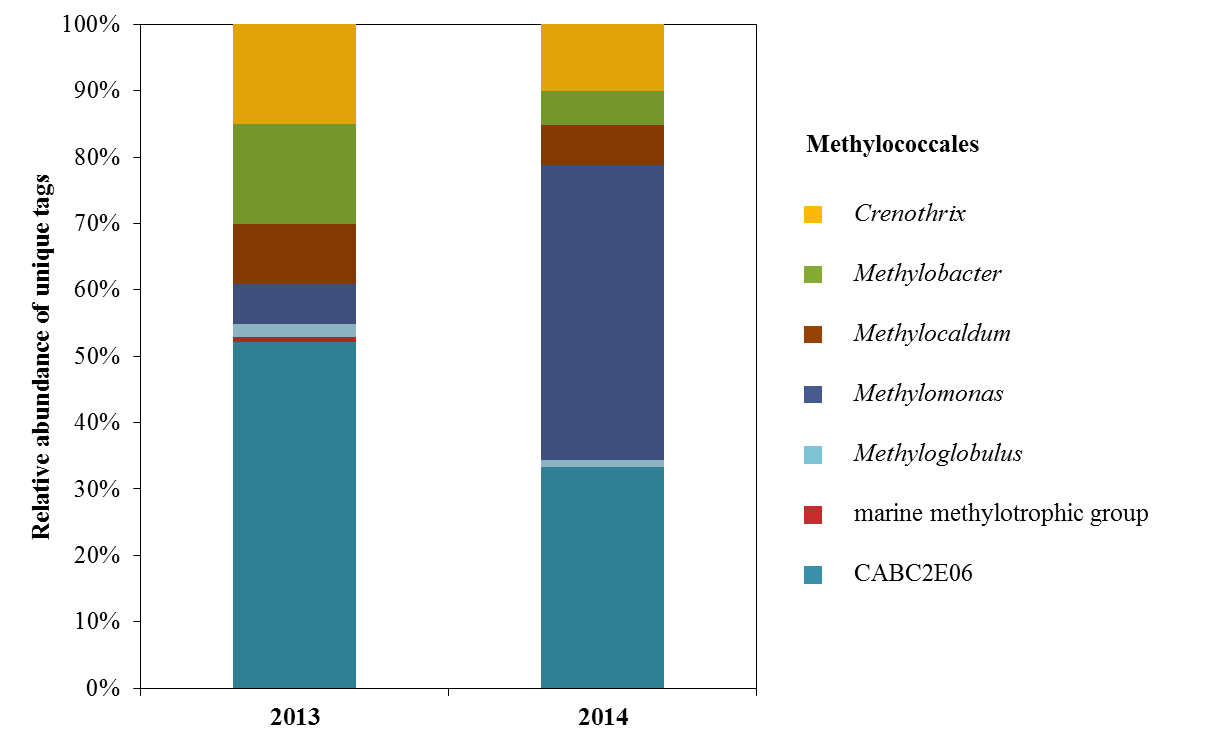
**

**Supplementary Figure 2. Taxonomic assignment of 16S rRNA gene amplicon sequences recovered from the Lake Rotsee oxycline** **in August 2013 and October 2014 assigned to the *Methylococcales* order (Bowman 2005).**

In this order, unicellular *Methylococcaceae*, CABC2E06 and *Crenothrix* 16S rRNA sequences comprised 31%, 52% and 15% (2013) and 56%, 33% and 10% (2014), respectively. Retrieved *Crenothrix* 16S rRNA sequences (n=66 and n= 270 for 2013 and 2014, respectively) comprised between 0.06-0.1% of all retrieved 16S rRNA bacterial sequences.

**
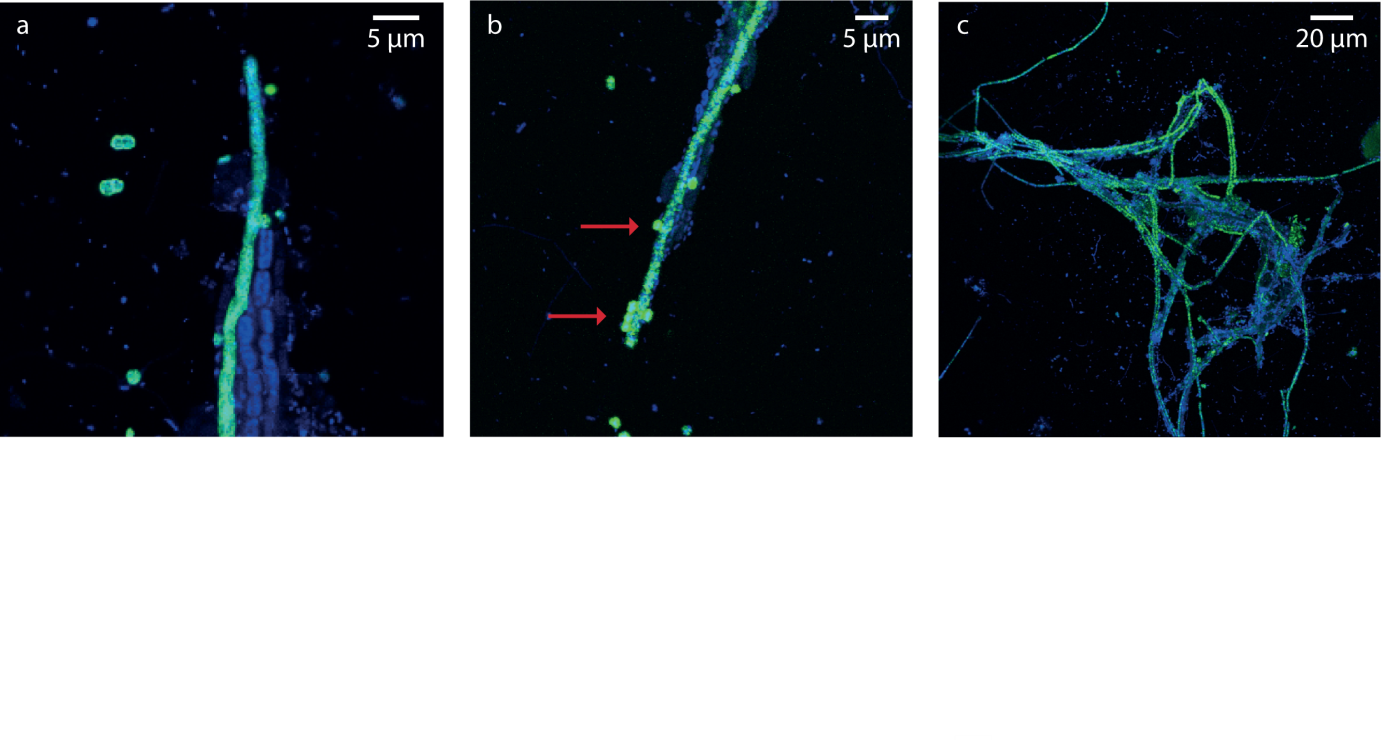
**

**Supplementary Figure 3. *Crenothrix* in Lake Rotsee.**

DAPI (blue) and CARD-FISH (green) signals of *Crenothrix* **a**, in the *in situ* samples from 8 m depth in 2014 (probe Mgamma669); **b**, at the beginning of the incubation experiment with water from 9 m depth in 2013 (probe Creno445). Red arrows indicate possible gonidial cells. **c**, Filaments after 11 days of oxic incubation under light conditions (probe Creno445).


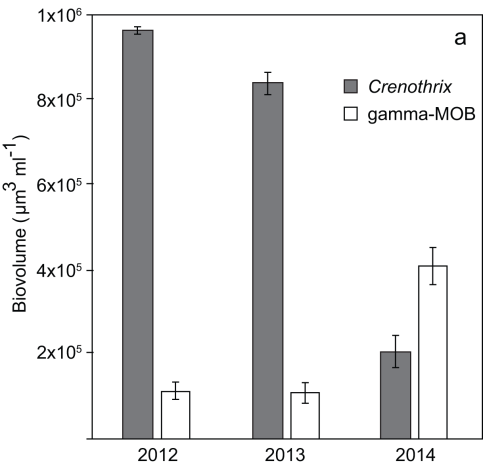

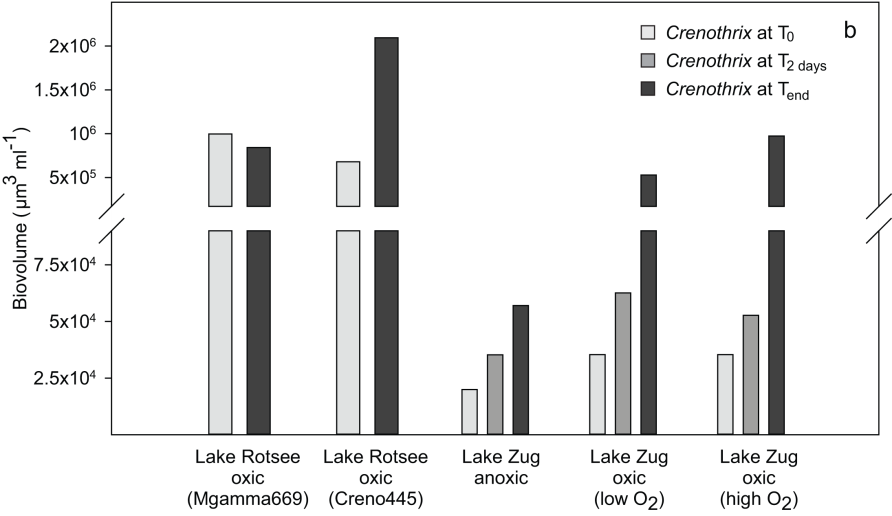


**Supplementary Figure 4. Overview of *Crenothrix* biovolume *in situ* and in the incubations.**

**a,** biovolume of *Crenothrix* filaments and unicellular gamma-MOB at and below the oxycline of Lake Rotsee. *Crenothrix* biovolume refers to an average biovolume determined for Mgamma669 and Creno445-hybridized filaments and gamma-MOB refers to an average biovolume determined for cells targeted by the Mgamma84+705 probes. Biovolume was calculated in depths that displayed the highest methane oxidation rates. In 2012 and 2014 this was below the oxycline (at 7 m depth) and in 2013 directly at the oxycline (at 9 m depth). Error bars represent a cumulative standard error of the mean between measured filaments and counted fields of view (n=20). **b**, Increase of *Crenothrix* filament biovolumes in oxic and anoxic incubation from Lake Rotsee and Lake Zug over time. *Crenothrix* in Lake Rotsee were detected using either Mgamma669 or Creno445 probe, *Crenothrix* in Lake Zug was only detected using Mgamma669 probe. Biovolume was determined for incubations from the oxycline [9 m depth in Lake Rotsee (2013) and 160 m in Lake Zug (2013, 2014)]. T_end_ refers to 11 days for Lake Rotsee incubation, 16 days for Lake Zug anoxic incubation and 11 days for Lake Zug oxic incubations.

**
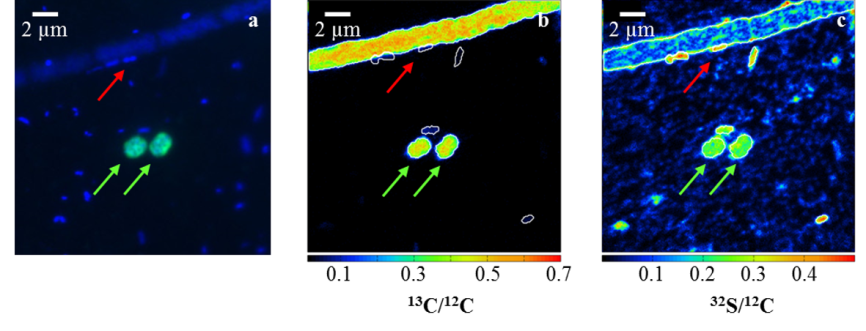
**

**Supplementary Figure 5. Methane-dependent growth of *Crenothrix* in Lake Rotsee.**

**a,** DAPI (blue) and CARD-FISH (green) fluorescent signals of gamma-MOB (green arrows, probes Mgamma84+705) and an unhybridized filamentous bacterium (red arrow) from Lake Rotsee oxic incubation. **b,** The corresponding ^13^C/^12^C nanoSIMS image shows uptake of ^13^CH_4_ by the coccoid gamma MOB as well as by the filamentous bacterium. Note that the ^13^C enrichment in the filamentous organism is comparable to that of the coccoid gamma-MOB. **c,** The corresponding ^32^S/^12^C nanoSIMS images show distribution of organic material in the analyzed fields of view. The higher S/C ratio of the unicellular gamma-MOB compared to *Crenothrix* cells might be due to a presence of the carbon-rich polysaccharide sheath surrounding the filaments.

**
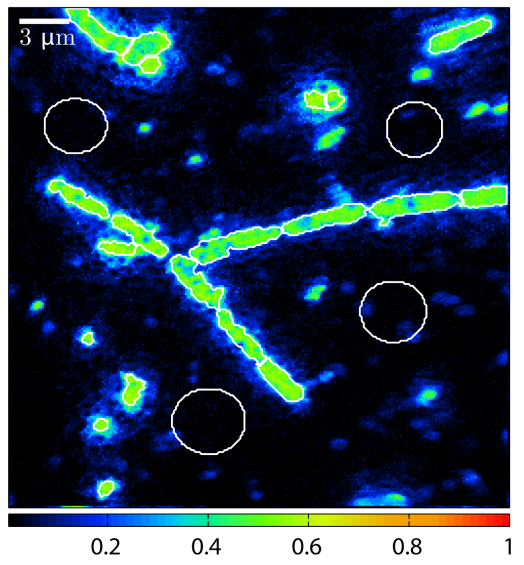
**

**Supplementary Figure 6. A ^12^C^15^N/^12^C^14^N nanoSIMS image corresponding to the field of view displayed in Figure 1g-i.**

Due to the long incubation time (16 days) it is not possible to conclude that the ^15^N in the cell biomass was taken up in the form of ^15^NO_3_^-^ which was added in the incubation. Therefore, the cellular ^15^N uptake is only used as an indicator of growth under incubation conditions.

**a**

**
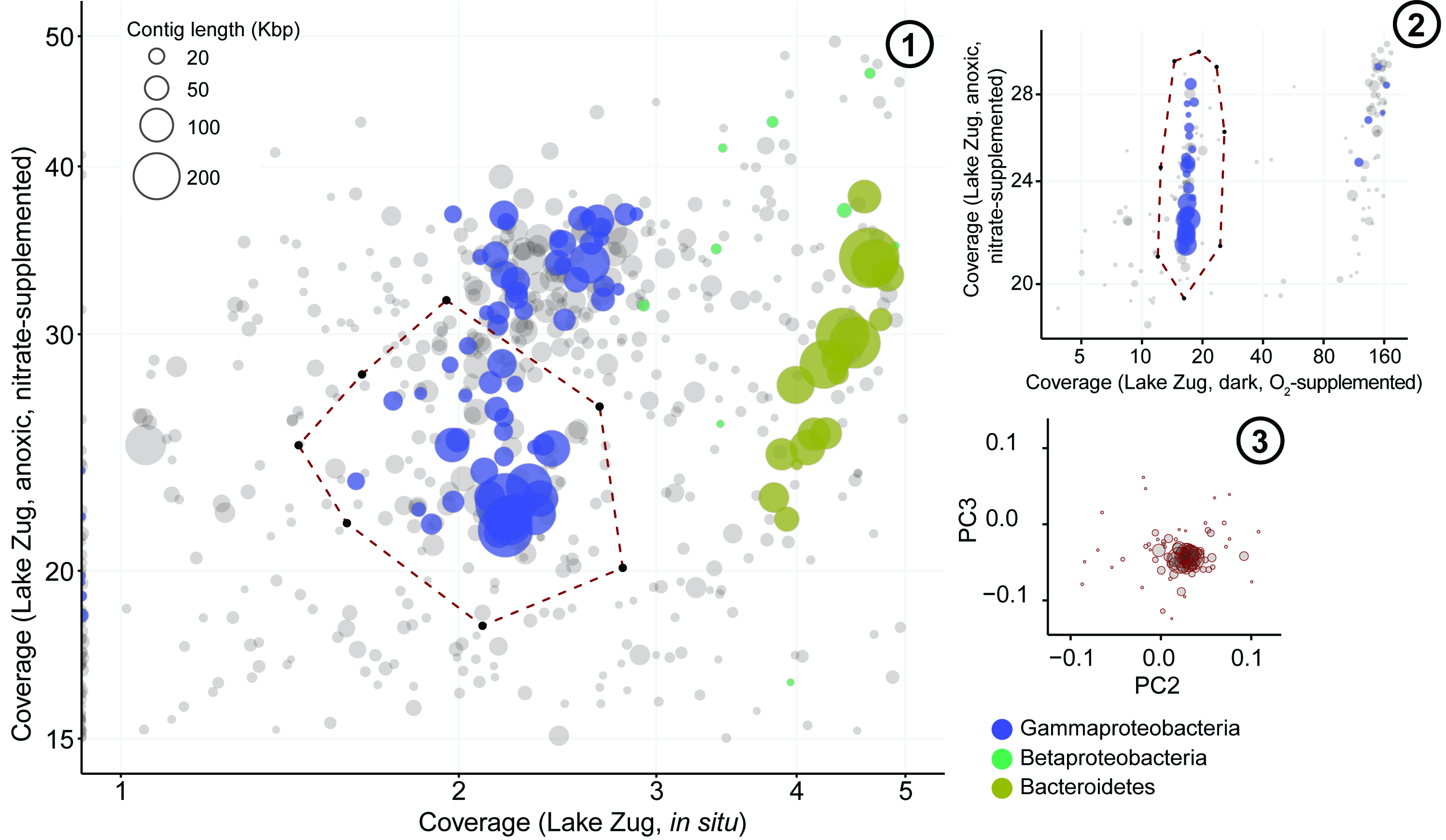
**

**b**

**
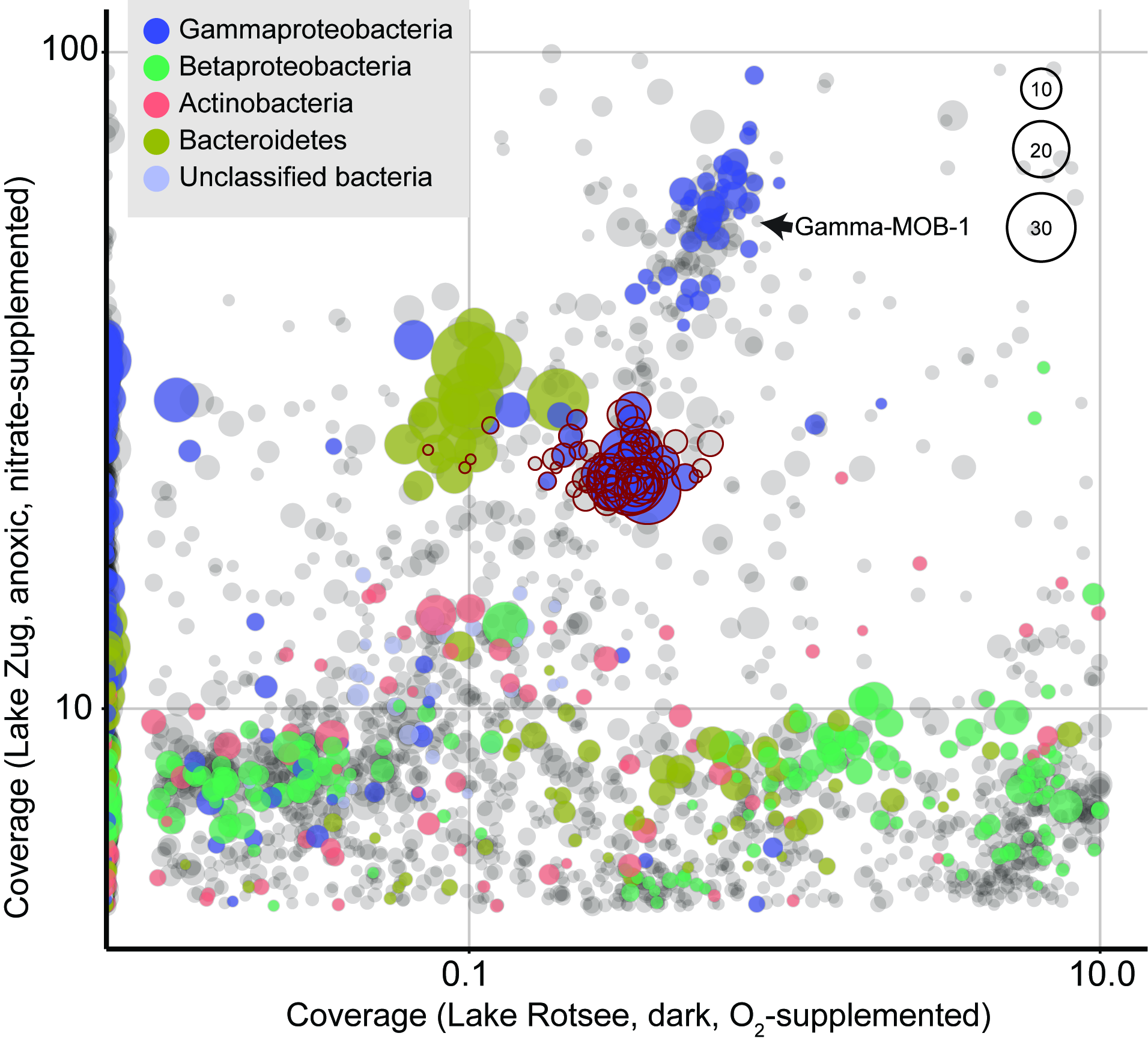
**

**Supplementary Figure 7. Differential coverage overview of the different bins retrieved from Lake Zug and their classification based on marker genes and 16S rRNA genes.**

**a,** Lacustrine *Crenothrix* D3 bin (outlined by dotted line) was extracted from metagenomic contigs of the Lake Zug assembly (metagenome data set Z3: Lake Zug, anoxic, nitrate-supplemented incubation) by exploiting differential coverage binning. Each contig is represented by a circle and the circles size reflects contig length (in Kbp). Colored circles show taxonomic assignment of essential single copy genes present on the contig. A preliminary bin was obtained by differential coverage of the Lake Zug *in situ* sample (data set: Z1) and the Lake Zug anoxic, nitrate-supplemented incubation (data set Z3; plot 1). This bin was further refined by differential coverage of the Lake Zug anoxic, nitrate-supplemented incubation (data set: Z1) and Lake Zug dark, O_2_-supplemented (data set: Z2, plot 2). Tetranucleotide frequencies of the final *Crenothrix* D3 bin are shown in plot 3. **b**, Differential coverage plot of the same Lake Zug assembly. Contigs are plotted with their respective average coverage of two sequenced samples: Lake Zug, anoxic, nitrate-supplemented incubation (data set: Z3; y-axis) as well as Lake Rotsee, dark, O_2_-supplemented incubation (data set: R2; x-axis). Lacustrine *Crenothrix* D3 bin (dark red circles) as well as another gamma-proteobacterial bin (Gamma-MOB-1) is shown.

**
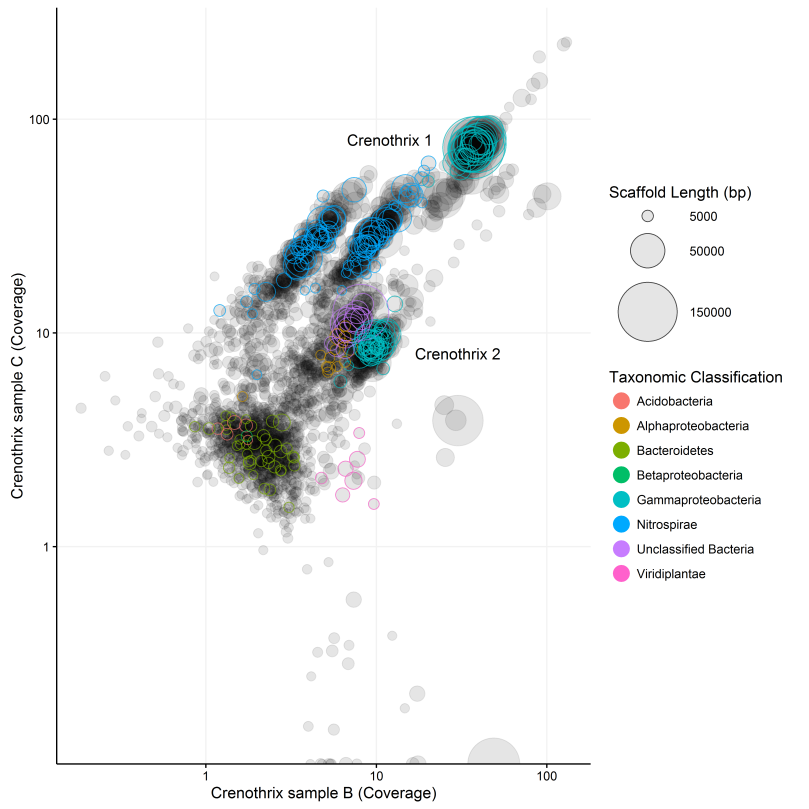
**

**Supplementary Figure 8: Differential coverage plot for the *C. polyspora* metagenomes.**

Differential coverage plot of the Wolfenbüttel waterworks sand filter (sieved *Crenothrix* biomass) assembly. Contigs are plotted with their respective average coverage of two sequenced samples: sample B, collected in 2005 and incubated at different methane concentrations for 24 hours (x-axis) as well as sample C, collected in 2004 and incubated with 500 μmol l^-1^ ammonium for 212 hours (y-axis). Each contig is represented by a circle and the circle size reflects contig length (in Kbp). Colored circles show taxonomic assignment of essential single copy genes present on the contig. Clusters of similarly colored circles represent potential genome bins. The two *Crenothrix* bins (light blue), two comammox *Nitrospira* bins (dark blue), and bins representing various other organisms (other colors) are shown.

**Supplementary References**

Bowman JP (2005). Order VII. *Methylococcales* ord. nov. In: Garrity G, Brenner DJ, Krieg NR, Staley JR (eds). *Bergey's manual of systematic bacteriology, 2nd ed.* Springer: New York, NY. pp 248-270.

Chistoserdova L (2011). Modularity of methylotrophy, revisited. *Environ Microbiol* **13:** 2603-2622.

Daims H, Bruhl A, Amann R, Schleifer KH, Wagner M (1999). The domain-specific probe EUB338 is insufficient for the detection of all Bacteria: Development and evaluation of a more comprehensive probe set. *Systematic and Applied Microbiology* **22:** 434-444.

Daims H, Lebedeva EV, Pjevac P, Han P, Herbold C, Albertsen M *et al.* (2015). Complete nitrification by Nitrospira bacteria. *Nature* **528:** 504-509.

Deutzmann JS, Hoppert M, Schink B (2014). Characterization and phylogeny of a novel methanotroph, *Methyloglobulus morosus* gen. nov., spec. nov. *Systematic and Applied Microbiology* **37:** 165-169.

Eller G, Stubner S, Frenzel P (2001). Group-specific 16S rRNA targeted probes for the detection of type I and type II methanotrophs by fluorescence in situ hybridisation. *Fems Microbiol Lett* **198:** 91-97.

Giovannoni SJ, Hayakawa DH, Tripp HJ, Stingl U, Givan SA, Cho JC *et al.* (2008). The small genome of an abundant coastal ocean methylotroph. *Environ Microbiol* **10:** 1771-1782.

Goris J, Konstantinidis KT, Klappenbach JA, Coenye T, Vandamme P, Tiedje JM (2007). DNA–DNA hybridization values and their relationship to whole-genome sequence similarities. *Int J Syst Evol Micr* **57:** 81-91.

Kalyuhznaya MG, Martens‐Habbena W, Wang T, Hackett M, Stolyar SM, Stahl DA *et al.* (2009). Methylophilaceae link methanol oxidation to denitrification in freshwater lake sediment as suggested by stable isotope probing and pure culture analysis. *Env Microbiol Rep* **1:** 385-392.

Khadem AF, Wieczorek AS, Pol A, Vuilleumier S, Harhangi HR, Dunfield PF *et al.* (2012). Draft genome sequence of the volcano-inhabiting thermoacidophilic methanotroph Methylacidiphilum fumariolicum strain SolV. *J Bacteriol* **194:** 3729-3730.

Konstantinidis KT, Tiedje JM (2005). Towards a genome-based taxonomy for prokaryotes. *J Bacteriol* **187:** 6258-6264.

Luo C, Rodriguez-R LM, Konstantinidis KT (2014). MyTaxa: an advanced taxonomic classifier for genomic and metagenomic sequences. *Nucleic Acids Res*.

Op den Camp HJ, Islam T, Stott MB, Harhangi HR, Hynes A, Schouten S *et al.* (2009). Environmental, genomic and taxonomic perspectives on methanotrophic Verrucomicrobia. *Env Microbiol Rep* **1:** 293-306.

Oswald K, Milucka J, Brand A, Littmann S, Wehrli B, Kuypers MMM *et al.* (2015). Light-dependent aerobic methane oxidation reduces methane emissions from seasonally stratified lakes. *Plos One* **10**.

Oswald K, Milucka J, Brand A, Hach P, Littmann S, Wehrli B *et al.* (2016). Aerobic gammaproteobacterial methanotrophs mitigate methane emissions from oxic and anoxic lake waters. *Limnol Oceanogr***:** n/a-n/a.

Palomo A, Jane Fowler S, Gulay A, Rasmussen S, Sicheritz-Ponten T, Smets BF (2016). Metagenomic analysis of rapid gravity sand filter microbial communities suggests novel physiology of Nitrospira spp. *The ISME Journal*.

Parks DH, Imelfort M, Skennerton CT, Hugenholtz P, Tyson GW (2015). CheckM: assessing the quality of microbial genomes recovered from isolates, single cells, and metagenomes. *Genome research* **25:** 1043-1055.

Pinto AJ, Marcus DN, Ijaz UZ, Bautista-de lose Santos QM, Dick GJ, Raskin L (2016). Metagenomic evidence for the presence of comammox Nitrospira-like bacteria in a drinking water system. *mSphere* **1**.

Pol A, Barends TR, Dietl A, Khadem AF, Eygensteyn J, Jetten MS *et al.* (2014). Rare earth metals are essential for methanotrophic life in volcanic mudpots. *Environ Microbiol* **16:** 255-264.

Richter M, Rosselló-Móra R (2009). Shifting the genomic gold standard for the prokaryotic species definition. *Proceedings of the National Academy of Sciences* **106:** 19126-19131.

Stoecker K, Bendinger B, Schoning B, Nielsen PH, Nielsen JL, Baranyi C *et al.* (2006). Cohn's Crenothrix is a filamentous methane oxidizer with an unusual methane monooxygenase. *P Natl Acad Sci USA* **103:** 2363-2367.

van Kessel MAHJ, Speth DR, Albertsen M, Nielsen PH, Op den Camp HJM, Kartal B *et al.* (2015). Complete nitrification by a single microorganism. *Nature* **528:** 555-559.

Varghese NJ, Mukherjee S, Ivanova N, Konstantinidis KT, Mavrommatis K, Kyrpides NC *et al.* (2015). Microbial species delineation using whole genome sequences. *Nucleic Acids Res*.

Wilson SM, Gleisten MP, Donohue TJ (2008). Identification of proteins involved in formaldehyde metabolism by Rhodobacter sphaeroides. *Microbiology+* **154:** 296-305.
